# pH controlled assembly of a self-complementary halogen-bonded dimer†
†Electronic supplementary information (ESI) available: Details of computational methods, synthesis and characterisation data for all compounds are provided. CCDC 1488032–1488035. For ESI and crystallographic data in CIF or other electronic format see DOI: 10.1039/c6sc03696a



**DOI:** 10.1039/c6sc03696a

**Published:** 2016-09-19

**Authors:** Leonardo Maugeri, Ellen M. G. Jamieson, David B. Cordes, Alexandra M. Z. Slawin, Douglas Philp

**Affiliations:** a School of Chemistry and EaStCHEM , University of St Andrews , North Haugh , St Andrews , Fife KY16 9ST , UK . Email: d.philp@st-andrews.ac.uk ; Fax: +44 1334 463808 ; Tel: +44 1334 467264

## Abstract

Phenols and their corresponding phenoxide anions can form halogen bonds with neutral iodotriazoles. The strength of these interactions depends critically on the protonation state of the oxygen atom – the interaction of the phenoxide anion is more than an order of magnitude stronger than the corresponding phenol. The assembly of a molecule bearing both an iodotriazole and a phenoxide anion into a self-complementary dimer, stabilised by two halogen bonds between the phenoxide anions and the neutral iodotriazoles has been demonstrated. The corresponding phenol shows no halogen bond mediated assembly either in the solid or in the solution state. This assembly process can be actuated simply by a change in protonation state – treatment of the phenol with one equivalent of base results in deprotonation and assembly of the dimer. The structure of the homodimer formed by the phenoxide-bearing iodotriazole has been determined in the solid state and ^19^F NMR spectroscopy demonstrates that the assembled dimer persists in solution and that it has significant stability. ^19^F NMR spectroscopy has also been used to demonstrate that the assembly process is completely reversible.

## Abstract 2

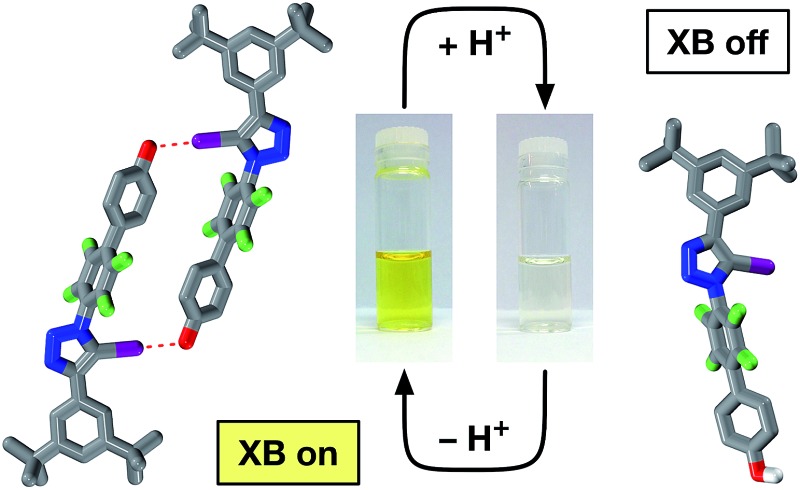
Halogen bonding between an oxygen acceptor and an iodotriazole donor can be switched on an off by cycling the solution pH.

## Introduction

Although first described more than a century ago, halogen bonds^1^ (XBs) – the noncovalent interaction between a Lewis acidic halogen atom and a Lewis base – have only recently been investigated as reliable tools^2^ for the assembly of complex molecular and supramolecular architectures. Led by the contributions of Metrangolo and Resnati from the late 1990s, to date, XBs have found applications in crystal engineering,^3^ medicinal chemistry,^4^ materials chemistry^5^ and nanoscience.^6^ The assembly of structures in solution using XB interactions in organic solvents constitutes a rapidly expanding^7^ area of research. These studies of XBs in solution are of pivotal importance for the understanding of the factors that contribute to the stability^8^ of an XB, whose understanding will ultimately lead to design rules for exploiting XBs that are on a similar footing to those available^9^ for hydrogen bonds. In addition, expanding the lexicon of XB-based interactions that are stable in solution is of significant importance for the realisation of XB-based supramolecular architectures^10^ that are persistent in solution. Previous reports concerning the stability of XB complexes formed between neutral organic XB donors, such as perfluorohalocarbons^8b,c^ (PFHCs) and aryl iodoacetylenes,^8*d*^ and a variety of neutral organic XB acceptors in organic solvents have demonstrated that the stability constants for such complexes are generally^11^ within the range of 0.1 to 10 M^–1^. The stability of these single point interactions is much too low to permit the formation of organised complex arrays. One strategy to enhance the halogen bonding ability of a XB donor is by rendering the interaction “charge assisted”, *i.e.* using a cationic XB donor. Examples of halopyridinium, haloimidazolium and halotriazolium XB donors have been implemented successfully in mechanically interlocked molecules by the group of Beer^10b–e^ for sensing applications and applied^12^ in catalysis by Huber and coworkers.

Using neutral organic XB donors, it is necessary to appeal to the concepts of interactional cooperativity^13^ to increase the stability of assemblies. A few research groups have managed to embed^10f–h^ aromatic PFHC XB donors within multivalent platforms that are able to interact with multivalent XB acceptors, forming neutral complexes, stabilised by multiple XBs, with stability constants in the range 10^3^ to 10^4^ M^–1^ in organic solutions.

We have become interested in applying our experience^14^ with self-complementary replicating templates to the creation of stable halogen-bonded assemblies in solution. Although halogen bond-based dimeric designs have been reported^15^ previously in the solid state, the formation of such constructs in solution remains elusive. Recently, we have demonstrated^16^ that a molecular scaffold incorporating a 5-iodo-1,4-diaryl-1*H*-1,2,3-triazole XB donor and a 3-oxypyridine XB acceptor and a 5-iodo-1,4-diaryl-1*H*-1,2,3-triazole XB donor can drive the formation of a halogen-bonded dimer in solution. The stability of this assembly was, however, disappointing and we reasoned that this molecular design could not exploit chelate cooperativity fully. Further, we reasoned that the XB interactions of pyridine acceptors were simply too weak to permit successful assembly of discrete dimeric structures in solution by aggregating only two XB interactions. In searching for an alternative XB acceptor, we were also intrigued by the possibility of exploiting an XB interaction that could be enabled and disabled by an external stimulus, such as a pH change.

## Results and discussion

The ability of phenols to function as XB acceptors as well as hydrogen bond (HB) donors has been widely investigated^17–19^ in crystal engineering. In fact, this dual character has been exploited in the realisation of crystal lattices featuring intricate networks of orthogonal HB and XB interactions. Phenoxide anions, on the other hand, despite the loss of their HB donating character, have been shown^20^ to be stronger XB acceptors than their neutral analogues as a result of their anionic character. Nevertheless, reports of phenoxide-based XB interactions in solution are scarce.

**Fig. 1:**
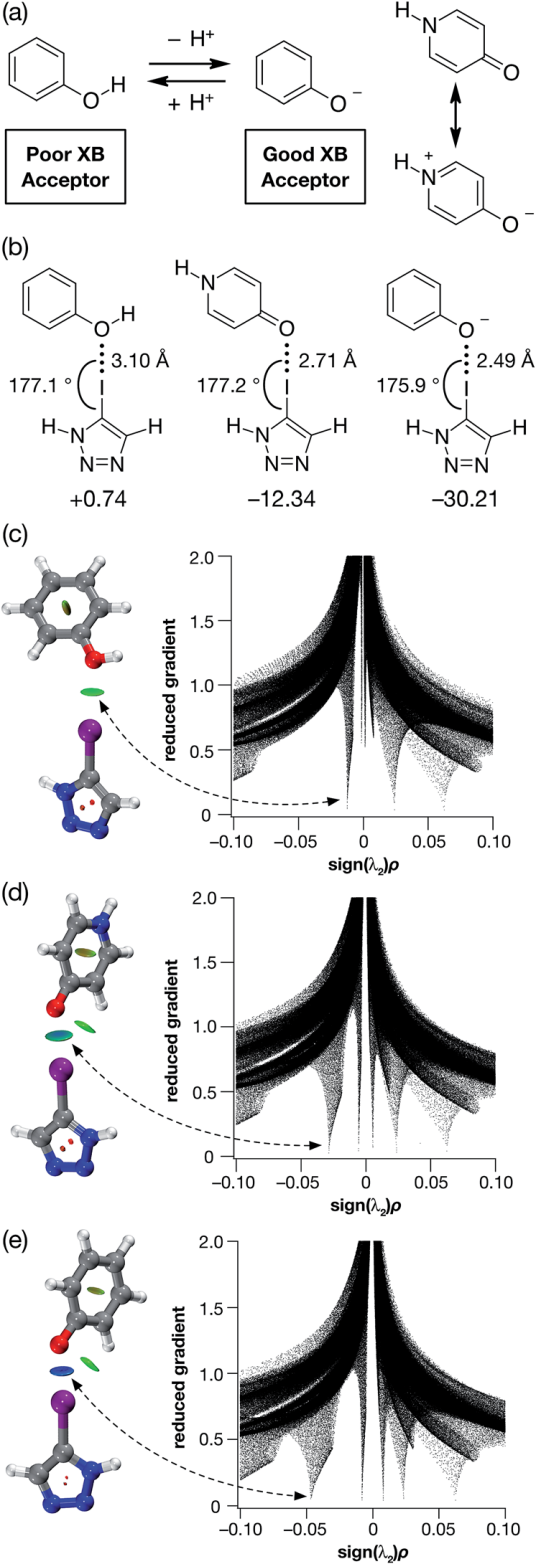
(a) The phenol-phenoxide acid–base pair as pH responsive halogen bond donors. (b) Calculated O···I geometries and interaction energies (TPSSh/def2-TZVP in acetonitrile (PCM model), enthalpies of complexation at 298 K in kJ mol^–1^) for the complexes between phenol, 4-pyridone and the phenoxide anion and 5-iodo-1*H*-1,2,3-triazole. Visualisation of the non-covalent interactions between 5-iodo-1*H*-1,2,3-triazole and (c) phenol, (d) 4-pyridone and (e) phenoxide anion. Left: Intermolecular interaction isosurfaces generated by NCIPLOT^22^ for *s* = 0.5 and –0.05 < sign(*λ*_2_)*ρ* < 0.05 (colour scale: attractive (blue) → repulsive (red)). Right: Plots of sign(*λ*_2_)*ρ vs.* reduced gradient highlighting the favourable interaction corresponding to the halogen bond at sign(*λ*_2_)*ρ* ∼ –0.035. Atom colouring: H atoms = white, C atoms = grey, N atoms = blue, O atoms = red, I atoms = purple.

Given their different XB accepting properties, we reasoned that the phenol-phenoxide acid–base pair offered^21^ (Fig. 1a) the ideal opportunity to create an XB interaction that could be switched on and off using a change in pH. We also identified the possibility that 4-pyridone could provide an alternative, neutral XB acceptor with similar properties to phenoxide by virtue of its charged resonance form (Fig. 1a).

In order to probe the potential of these three acceptors to form XBs, we performed a series of calculations on the complexes formed in acetonitrile solution between phenol, 4-pyridone and the phenoxide anion and 5-iodo-1*H*-1,2,3-triazole at the TPSSh/def2-TZVP level of theory. The geometries and stabilities of these complexes (Fig. 1b) demonstrate the gradation in behaviour from phenol, through 4-pyridone, to the phenoxide anion. In the case of phenol, the O···I contact is very long (3.10 Å) and the enthalpy of complexation at 298 K is essentially zero at 298 K, indicating that phenol would be incapable of functioning as an XB donor in solution.

By contrast, the phenoxide anion forms an extremely short O···I contact with the iodotriazole and the complex is predicted to be very stable. As expected, 4-pyridone lies between these two extremes. Analysis of the reduced gradient^22^ of the electron density (Fig. 1c–e) reveals the changing nature of these interactions. The low density, low gradient region associated with the phenol·iodotriazole complex is van der Waals-like in nature and is barely attractive. By contrast, the low density, low gradient regions associated with the other two complexes are both significantly attractive – particularly in the case of the phenoxide anion (sign(*λ*_2_)*ρ* ∼ –0.05).

**Fig. 2:**
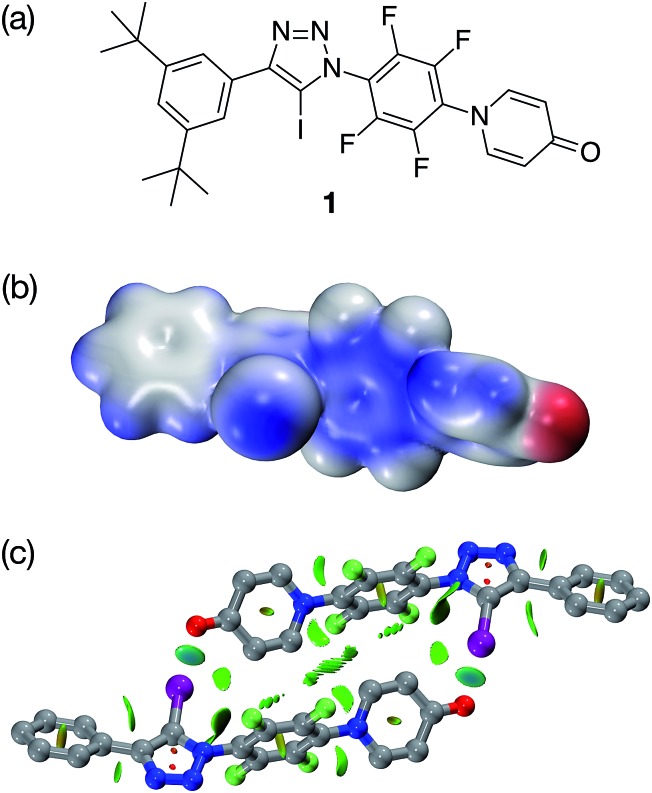
(a) Design of a self-complementary iodotriazole. (b) Electrostatic potential surface of **1** (TPSSh/def2-TZVP). Colour scale: blue = positive → red = negative (c) Calculated structure of the [**1**·**1**] including a visualisation of the non-covalent interactions present in the dimer. Intermolecular interaction isosurfaces generated by NCIPLOT^22^ for *s* = 0.5 and –0.05 < sign(*λ*_2_)*ρ* < 0.05 (colour scale: attractive (blue) → repulsive (red)). XB geometry: I···O=C *r*(O···I) = 2.77 Å and ∠(O···I–C) = 173.3°. Atom colouring: H atoms = white, C atoms = grey, N atoms = blue, O atoms = red, I atoms = purple.

In order to validate these calculations, we focused initially on the XB capabilities of a 4-pyridone-based system. Accordingly, we designed (Fig. 2a) iodotriazole **1**. The potential stability of the [**1**·**1**] dimer was assessed computationally at the TPSSh/def2-TZVP level of theory. In order to reduce the computational cost, the *tert*-butyl groups present in **1** for solubility reasons were omitted from the calculated structures. These calculations reveal that the iodotriazole should possess (Fig. 2b) an electrostatic potential surface that is self-complementary – a significant area of positive charge is associated with the σ hole on the iodine atom and a significant area of negative charge is associated with the carbonyl oxygen atom of the pyridone ring.

The calculations also predict (Fig. 2c) that the iodotriazole is capable of forming an approximately centrosymmetric dimer characterised by two short I···O=C XB contacts – *r*(O···I) = 2.77 Å and ∠(O···I–C) = 173.3°. The calculated enthalpy for the formation of the dimer at 298 K (see ESI for details†) is –43.9 kJ mol^–1^. Analysis of the reduced gradient of the electron density reveals two low density, low gradient regions in the O···I internuclear gap associated with the halogen bonds (pale blue, Fig. 2c) and a region of weakly attractive, van der Waals-like interactions along the spine of the dimer (green, Fig. 2c). Further insight into the interactions between the two molecules **1** present in the dimer comes from an examination of a Natural Bond Orbital (NBO) analysis^23^ of the system. The sum of the second order perturbation energies for the interactions between the lone pairs located on the carbonyl oxygen atoms of the pyridine ring and the σ* orbital associated with the C–I bond in the iodotriazole in the dimer are 36.4 kJ mol^–1^, suggesting that this interaction is relatively important in stabilising the homodimer.

Iodotriazole **1** was prepared in high yield, starting from the appropriate perfluoroaryl iodotriazole and 4-hydroxypyridine in presence of anhydrous potassium carbonate in acetonitrile, using S_N_Ar methodology we have described^16^ previously (see ESI for details†).

**Fig. 3:**
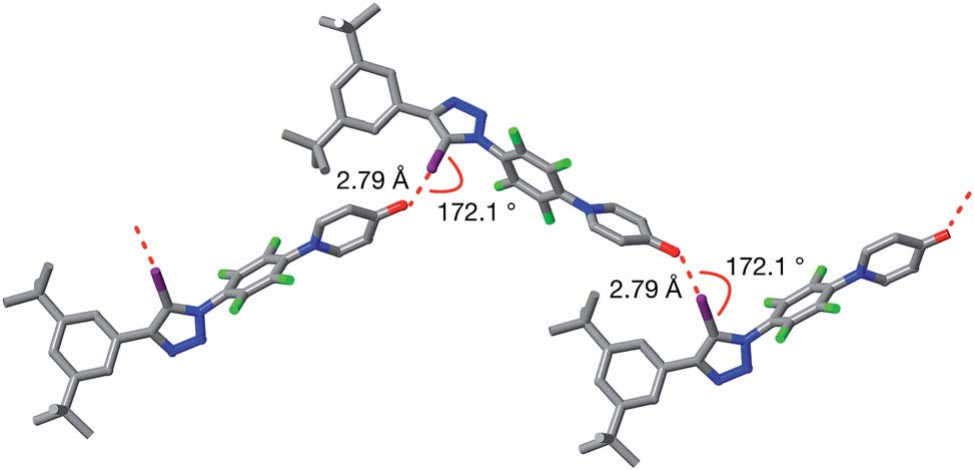
Solid state structure of **1** determined from single crystal X-ray diffraction data. Halogen bonds are marked as a red dashed lines (*r*(O···I) = 2.790 Å; ∠(O···I–C) = 172.1°). Atom colouring: C atoms = grey, N atoms = blue, O atoms = red, I atoms = purple. Hydrogen atoms and included solvent of crystallisation (toluene) are omitted for clarity.

Single crystals, suitable for analysis by X-ray diffraction, were obtained by slow evaporation of a saturated toluene solution of **1**. The solid state structure of **1** (Fig. 3) reveals chains of the self-complementary iodotriazole units connected by XB interactions (*r*(O···I) = 2.790 Å; ∠ (O···I–C) = 172.1°). Interestingly, the same crystal growth method afforded a second batch of crystals, which diffracted significantly more weakly than the first set. Although single crystal X-ray diffraction data were collected on these crystals, the data was not of sufficient quality to permit refinement of the structure solution to an acceptably low *R*% value. However, even with low quality data, it was clear that, within the solid state structure of these crystals, the self-complementary iodotriazole **1** formed a homodimeric assembly (see ESI for details†).

**Fig. 4:**
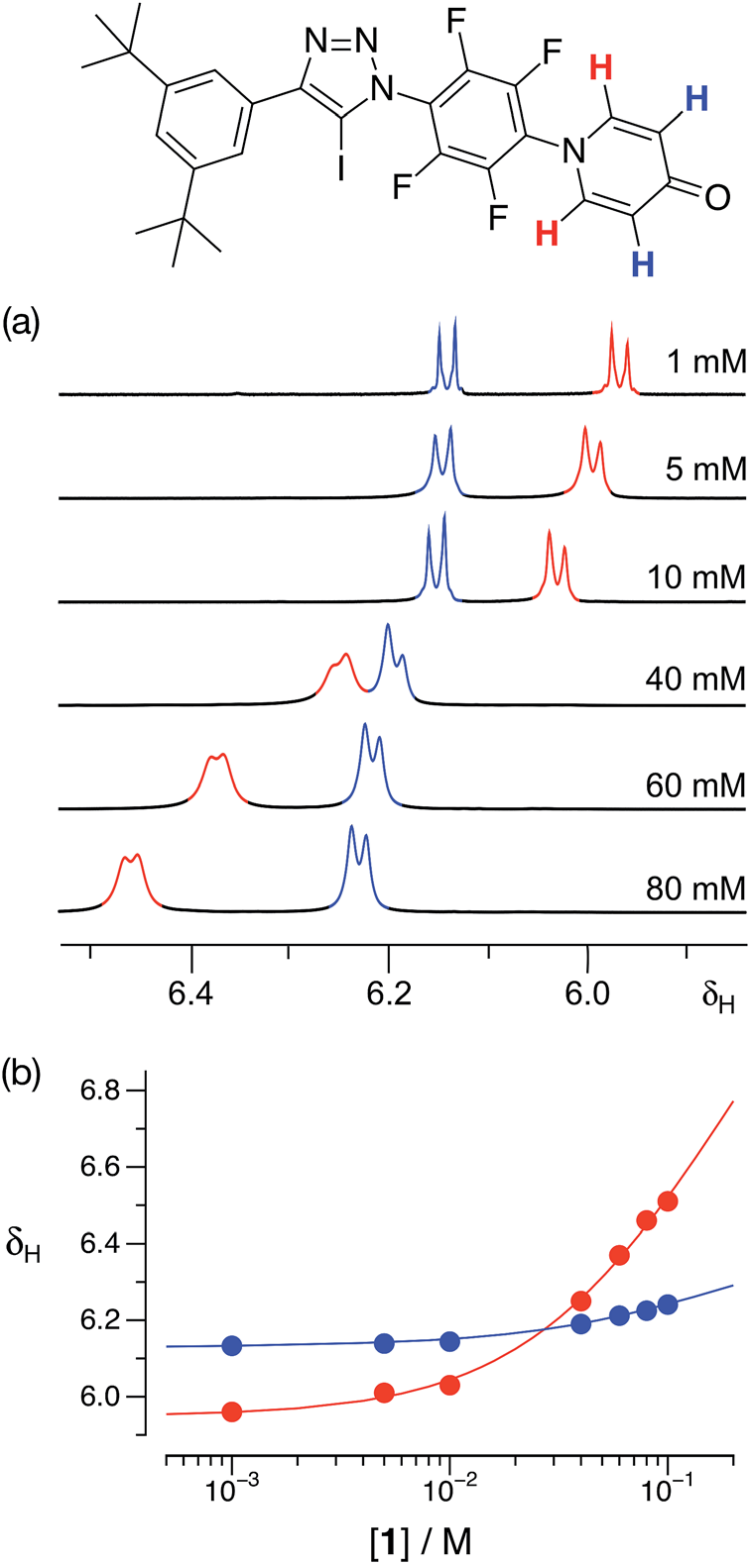
(a) Partial 500.1 MHz ^1^H{^19^F} NMR spectra of iodotriazole **1** at various concentrations in *d*_8_-toluene at 293 K showing the ^1^H resonances arising from the pyridone ring protons. (b) Best fits (solid lines) of the changes in the chemical shifts of the ^1^H resonances arising from the pyridone ring protons as a function of concentration to a **1** + **1** ⇄ [**1**·**1**] dimerisation model.

With some evidence in hand that **1** could form homodimers in the solid state, we wished to establish the ability of **1** to form homodimers in solution. Accordingly, we performed a dilution experiment using a solution of **1** in *d*_8_-toluene and a 500.1 MHz ^1^H{^19^F} NMR spectrum was recorded at a series of concentration steps – starting from an initial concentration of 80 mM, the solution of **1** was diluted progressively down to 1 mM. In this range of concentrations, the resonances arising from the pyridone ring protons of **1** experienced (Fig. 4a) significant chemical shift changes. The data from this dilution experiment for both probe protons can be fitted^24^ simultaneously (Fig. 4b) to a dimerisation binding model to afford a stability constant for the [**1**·**1**] dimer of 2.3 ± 0.3 M^–1^ in *d*_8_-toluene at 293 K corresponding to a free energy of dimerisation of –2.0 ± 0.3 kJ mol^–1^ at this temperature.

**Fig. 5:**
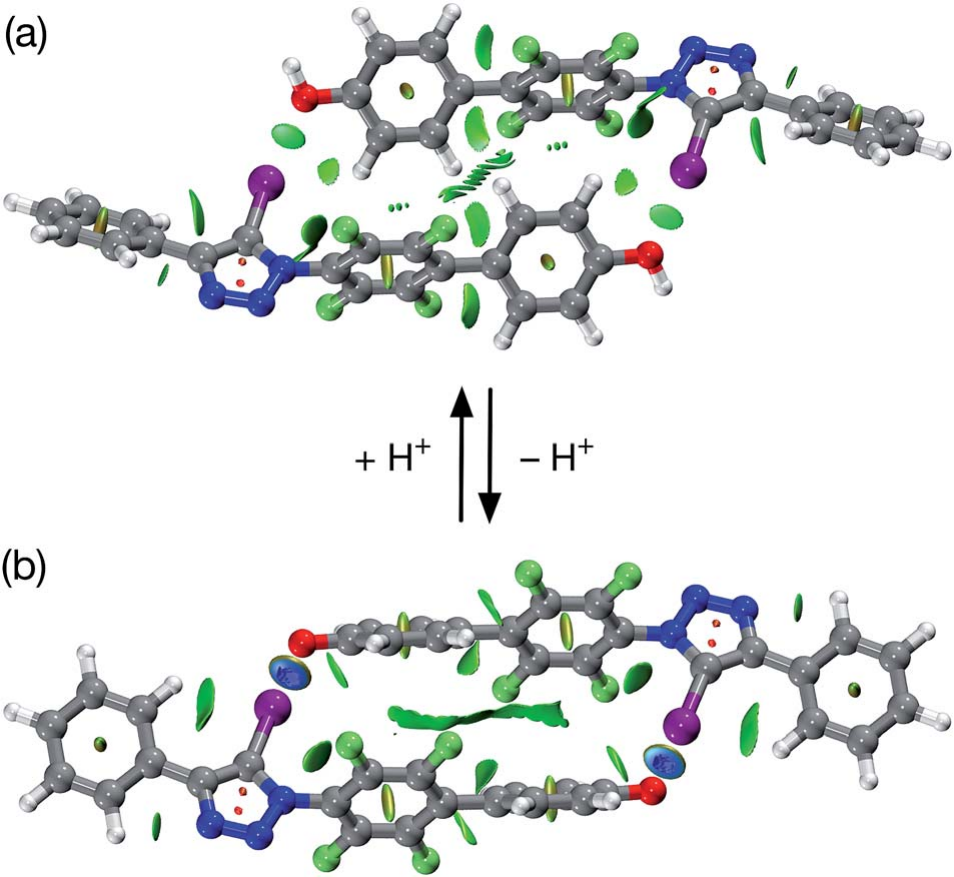
(a) Calculated structure of a putative dimer assembled from phenol bearing a diaryl iodotriazole. The structure includes a visualisation of the non-covalent interactions present. XB geometry: I···O=C *r*(O···I) = 3.15 Å and ∠(O···I–C) = 173.3°. (b) Calculated structure of a dimer assembled from the corresponding phenoxide anion, including a visualisation of the non-covalent interactions present. XB geometry: I···O=C *r*(O···I) = 2.51 Å and ∠(O···I–C) = 176.7°. In both cases, the non-covalent interactions present are visualised using intermolecular interaction isosurfaces generated by NCIPLOT^22^ for *s* = 0.5 and –0.05 < sign(*λ*_2_)*ρ* < 0.05 (colour scale: attractive (blue) → repulsive (red)). Atom colouring: H atoms = white, C atoms = grey, N atoms = blue, O atoms = red, I atoms = purple.

The results obtained for pyridone **1** provided encouragement that employing the phenol-phenoxide acid–base pair should provide a viable route to achieving our objective of a pH-switchable XB assembly. Accordingly, we investigated the system shown in Fig. 5 computationally. This system incorporates a diaryl iodotriazole XB donor and a phenol in an appropriate structural relationship in order to facilitate dimerisation. The calculated structure (TPSSh/def2-TZVP) of the putative phenol dimer (Fig. 5a) reveals an almost centrosymmetric homodimer in which there are two very long O···I contacts (*r*(O···I) = 3.15 Å, ∠(C–I···O = 174.9°)).

The calculated enthalpy of interaction at 298 K for this dimer is –4.26 kJ mol^–1^ at this level of theory, suggesting that any halogen bonding present in this dimer is not significantly stabilising. Analysis of the reduced gradient of the electron density (Fig. 5a) shows the characteristic low density, low gradient regions associated with weakly attractive interactions (green areas, Fig. 5a) both in the O···I internuclear gap and along the spine of the dimer. Further support for the relatively weak interactions between the two phenol molecules in the dimer came from an examination of a Natural Bond Orbital (NBO) analysis^23^ of the system. The sum of the second order perturbation energies for the interactions between the lone pairs located on the phenol oxygen atoms and the σ* orbital associated with the C–I bond in the iodotriazole in the dimer are only 10.3 kJ mol^–1^, well below that calculated for the pyridone-based [**1**·**1**] dimer.

Removal of a proton from the phenol affords the corresponding phenoxide anion (Fig. 5b) and, once again, we assessed the potential stability of the dimer formed by this anion at the TPSSh/def2-TZVP level of theory (Fig. 5b). In common with the phenol (Fig. 5a), the calculation reveals an almost centrosymmetric homodimer. In this case, however, there are two very short O···I contacts (*r*(O···I) = 2.51 Å, ∠(C–I···O = 176.7°)) present and the enthalpy of dimerisation at 298 K is predicted to be –50.4 kJ mol^–1^ at this level of theory – this value is only around 15% higher than the predicted enthalpy of dimerisation at 298 K for the [**1**·**1**] dimer. This observation is apparently at odds with the computational results obtained for the simple iodotriazole complexes shown in Fig. 1. In this series of simple 1 : 1 complexes, the enthalpy of complexation rises steeply as the XB acceptor changes from phenol through 4-pyridone to the phenoxide anion. However, dimer [**2**·**2**]^2–^ suffers from strong electrostatic destabilisation as a result of the anionic nature of the two partners in the dimer. Since no counterions are included in the calculation, this effect is not mitigated in any way and the value obtained for the enthalpy of dimerisation should be viewed as a worst case value.

Analysis of the reduced gradient of the electron density (Fig. 5b) shows the characteristic low density, low gradient regions in the O···I internuclear gap associated with significantly attractive halogen bonds (blue areas, Fig. 5b, sign(*λ*_2_)*ρ* ∼ –0.05). In common with the pyridone dimer [**1**·**1**], there are other, weaker, attractive interactions along the length of the dimeric structure. NBO analysis of this system reveals a second order perturbation energy for the interaction between the lone pairs located on the phenoxide oxygen atom and the σ* orbital associated with the C–I bond in the iodotriazole of 91.9 kJ mol^–1^, which contrasts starkly with that for the corresponding phenol dimer and is indicative of the much stronger association between the two anionic species.

In order to test these theoretical predictions, we synthesised phenol **2**–H using standard methods (see ESI for details†). Crystals of **2**–H, suitable for analysis by X-ray diffraction, were grown by slow evaporation of a solution of the phenol in THF. The solid state structure of **2**–H reveals (see ESI†) that, as expected, there is no evidence of halogen bonds formed between the phenolic oxygen atom and the iodotriazole. The structure contains one solvent molecule (THF) of crystallisation and the oxygen atom of the THF molecule is hydrogen bonded to the phenolic proton. Progressive dilution of a CD_3_CN solution of **2**–H from 20 mM to 1 mM did not result in any measurable chemical shift changes in the ^19^F resonances arising from the perfluorinated ring of **2**–H (see ESI†). These data suggest that **2**–H is unable to undergo XB-mediated dimerisation in solution in this concentration range.

**Fig. 6:**
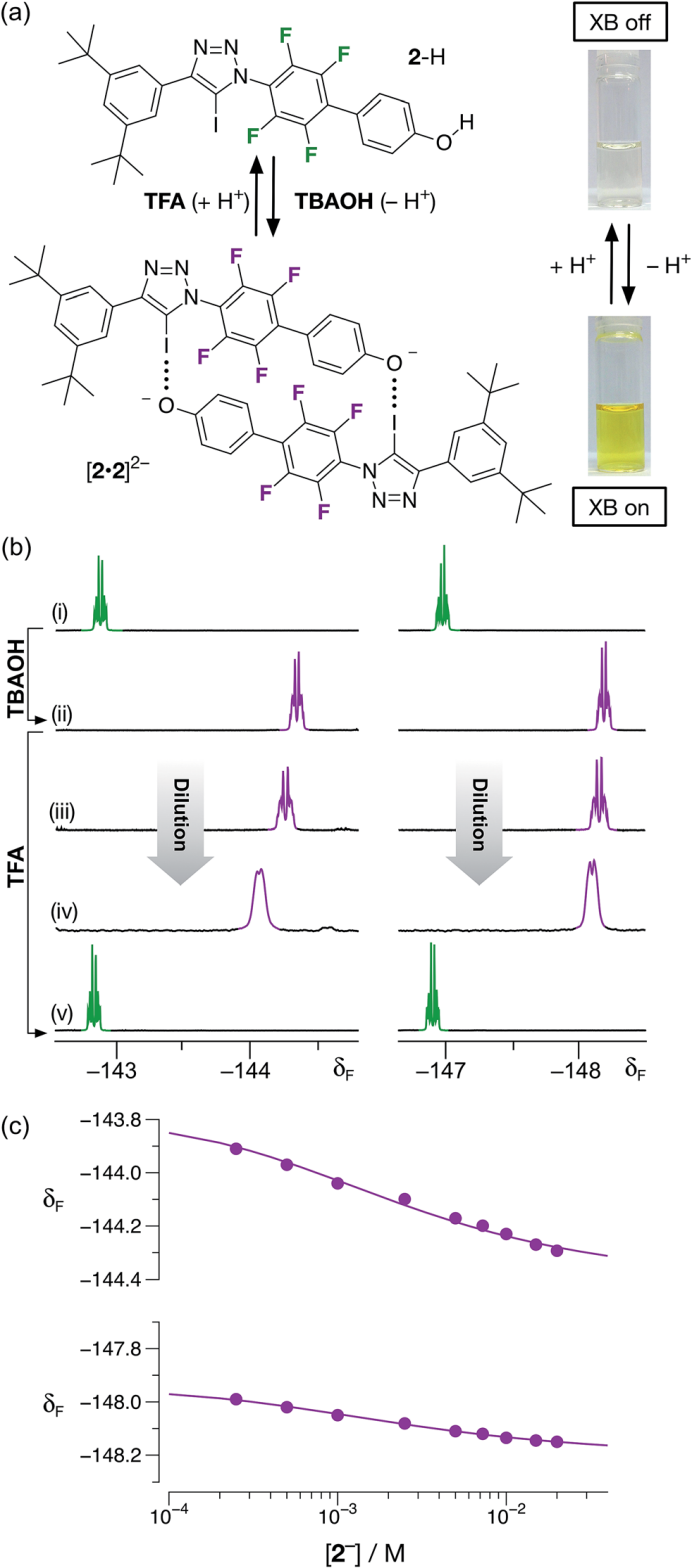
(a) The monomer/dimer state of **2** can be controlled by changing the protonation state of the phenolic oxygen atom by addition of **TBAOH** or **TFA**. (b) Partial 470.6 MHz ^19^F{^1^H} NMR spectra (298 K, CD_3_CN) of **2**: (i) 20 mM before addition of **TBAOH**; (ii) 20 mM after addition of **TBAOH**; (iii) 7.5 mM after addition of **TBAOH**; (iv) 1 mM after addition of **TBAOH**; (v) after treatment of 20 mM solution (ii) with TFA. (c) Best fits (solid lines) of the changes in the chemical shifts of the ^19^F resonances at *δ* –144 and –148 as a function of concentration to a **2** ^–^ + **2** ^–^ ⇄ [**2**·**2**]^2–^ dimerisation model.

Having demonstrated that phenol **2**–H was incapable of participating in halogen bonds under the conditions employed, we next turned to the potential of the corresponding phenoxide anion **2** ^–^ to form a halogen-bonded dimer. Treatment of a colorless, 20 mM solution of **2**–H in CD_3_CN at room temperature with one equivalent of a 1 M solution of tetrabutylammonium hydroxide (**TBAOH**) in methanol resulted in the development of an intense yellow color (Fig. 6a) in the solution, consistent with the formation of the corresponding phenoxide anion through deprotonation of **2**–H.

The formation of the anion **2** ^–^ was confirmed by comparison of the 470.6 MHz ^19^F{^1^H} NMR spectrum of the solution with **TBAOH**. After the addition of the base, the two resonances corresponding to aryl fluorine atoms at *δ* –142.7 and *δ* –146.9 are replaced two new resonances significantly upfield from those associated with **2**–H, at *δ* –144.3 and –148.2, respectively. This observation is consistent with the formation of the phenoxide anion. A freshly-prepared solution of **2** ^–^, as its tetrabutyl ammonium (**TBA**) salt, in CD_3_CN was diluted progressively (Fig. 6b(ii) → (iv)) from 20 mM to 1 mM. Downfield chemical shift changes (Δ*δ* = +0.38 and +0.16 between 20 mM and 1 mM) of the two resonances arising from the aryl fluorine atoms are observed, commensurate with the presence of the [**2**·**2**]^2–^ dimer. Simultaneous fitting^24^ (Fig. 6c) of the chemical shift changes for the two ^19^F resonances at around *δ* –144 and *δ* –148 to a dimerisation binding model affords a stability constant for the [**2**·**2**]^2–^ dimer of 510 ± 30 M^–1^, corresponding to a free energy of complexation of –15.2 ± 0.2 kJ mol^–1^ at 293 K. In order to assess the stability of the [**2**·**2**]^2–^ homodimer in the context of both chelate cooperativity and any electrostatic repulsion experienced within [**2**·**2**]^2–^, we sought to measure experimentally the single point association of a phenoxide anion with a suitable diaryl iodotriazole. Unfortunately, this measurement proved to be remarkably difficult to perform. Attempts to titrate a solution of tetrabutylammonium phenolate into a model iodotriazole in CD_3_CN at 293 K (see ESI for details†) resulted in rapid conversion of the 5-iodotriazole to the corresponding 5-*H* triazole. This reactivity is not observed with the dimeric species [**2**·**2**]^2–^ under the same conditions. Other laboratories have described^25^ situations in which 5-iodotriazoles undergo formal reductive deiodination to afford their 5-*H* analogues. However, we were able to perform a titration successfully when the phenolate solution was prepared using 1,8-diaza[5.4.0]undec-7-ene (**DBU**) as the base. This titration (see ESI for details†) afforded an association constant (*K*_a_) for the single point association between the phenolate anion and the model diaryliodotriazole of 6.6 ± 0.2 M^–1^. This interaction seems surprisingly weak and it is necessary to interpret the measured stability constant with caution. The phenolate counterion – **DBU**H^+^ – is capable of hydrogen bonding to the phenoxide oxygen atom reducing its ability to function as a halogen bond acceptor. However, this value can be used to place an upper limit on the level of cooperativity present within the [**2**·**2**]^2–^ homodimer – the connection free energy^26^ has an upper limit of around 6 kJ mol^–1^ and the effective molarity^13^ for the formation of the [**2**·**2**]^2–^ homodimer has an upper limit of around 10 M.

All attempts to isolate and crystallise the tetrabutyl ammonium salt of the [**2**·**2**]^2–^ dimer from CD_3_CN solutions were unsuccessful, principally as a result of the hygroscopic character of this salt. However, treatment of a concentrated solution of **2**–H in CD_3_CN with 15 equivalents of 1,8-diaza[5.4.0]undec-7-ene (**DBU**) (see ESI for details†) led to the spontaneous formation of crystals of [**2**·**2**]·(**DBU**H)_2_ suitable for analysis by single crystal X-ray diffraction.

**Fig. 7:**
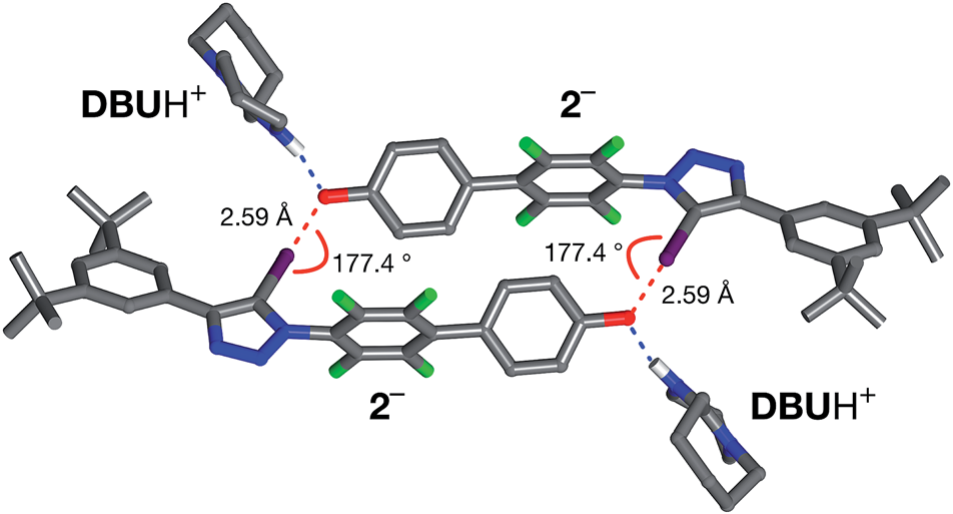
Solid state structure of the salt formed by reaction of **2** with 1,8-diaza[5,4,0]undec-7-ene (**DBU**) determined from single crystal X-ray diffraction data. Halogen bonds are marked as a red dashed lines (*r*(O···I) = 2.592 Å; ∠(O···I–C) = 177.2°). Hydrogen bonds from [**2**·**2**]^2–^ to the **DBU**H^+^ cations are shown as dashed blue lines. Atom color code: C, grey; N, blue; O, red; F, green; I, purple. Most hydrogen atoms are omitted for clarity.

The solid state structure of these crystals, determined from diffraction data, reveals (Fig. 7) a halogen-bonded homodimeric structure for [**2**·**2**]^2–^ in which the individual phenoxide anions are connected by remarkably short (*r*(O···I) = 2.592 Å, ΣvdW radii = 3.55 Å) and linear (∠(C–I···O = 177.2°)) I···O contacts. There is excellent agreement between structure of [**2**·**2**]^2–^ determined in the solid state and the calculated structure (Fig. 5). Additionally, there is a hydrogen bond from each of the **DBU**H^+^ cations to an oxygen atom in the corresponding phenoxide anion.

## Conclusions

In order for halogen bonds to achieve the status of a robust structure-directing element in the design of solution phase assemblies, it is necessary for halogen bonds to demonstrate the same level of flexibility and programmability as the ubiquitous hydrogen bond. Single hydrogen bonds are rarely used as a structure-directing element in the design of non-covalent assemblies – more commonly, several are used in concert to create stable structures. The aggregation of multiple hydrogen bonds in arrays are subject to clear design rules. The assembly and disassembly of the [**2**·**2**]^2–^ dimer in solution, actuated by proton transfer, represents an important step forward in the use of halogen bonds in this context. Not only does the [**2**·**2**]^2–^ dimer have significant stability in solution, its design was reached in a rational manner obeying clear design rules. Additionally, although Schöllhorn and coworkers have recently demonstrated^27^ that both the XB accepting and donating character of electrochemically active molecules can be modulated by the applied potential, the work presented here is, to the best of our knowledge, the first time that proton transfer has been exploited efficiently to regulate the formation of a XB-based assembly. However, in order to fully exploit the strong halogen bonds formed by phenoxide anions with aryl iodides, such as the iodotriazole used here, it is necessary to overcome the electrostatic repulsion experienced within the [**2**·**2**]^2–^ homodimer. This objective requires the dimer to be zwitterionic in its active (assembled) state and could be accomplished readily by switching^10d,e^ the XB donor to iodotriazolium. These studies, in the context of supramolecular materials based on the design of the [**2**·**2**]^2–^ homodimer are currently underway in our laboratory.
