# Supplementary material for: pH controlled assembly of a self-complementary halogen-bonded dimer
Source: Chem Sci. 2016 Sep 19;8(2):938–45. doi: 10.1039/c6sc03696a (PMC5452264; doi:10.1039/c6sc03696a)
Supplement: Supplementary file 1 [file SC-008-C6SC03696A-s001.pdf]

Electronic Supplementary Material (ESI) for Chemical Science.
This journal is © The Royal Society of Chemistry 2016

Electronic Supporting Information

pH Controlled assembly of a self-complementary halogen bonded dimer

Leonardo Maugeri, Ellen M. G. Jamieson, David B. Cordes,
Alexandra M. Z. Slawin, and Douglas Philp*

*School of Chemistry and EaStChem, University of St Andrews, North Haugh,
St Andrews, Fife KY16 9ST (UK).*

Table of contents

S1. Reagents and general equipment 3

S2. Synthetic procedures 4

Synthesis of 1-(4-(4-(3,5-di-tert-butylphenyl)-5-iodo-1*H*-1,2,3-triazol-1-yl)-2,3,5,6-tetrafluoro phenyl)pyridin-4(1*H*)-one (**1**) 4

Synthesis of phenol-appended iodotriazole **2**-H 5

S3. Solid state structures of **1** and solution state studies 8

S4. Crystal structures of iodotriazoles **S3** and **2**-H 9

S5. Deprotonation of phenol **2**-H and related control experiments 11

S6. Single point XB association constant determination 13

S7. Preparation of [**2**•**2**]•(**DBU**H)$_2$ 15

S8. DFT calculations 16

S9. References 17

S10. Crystallographic Informations 19

S11. NMR Spectra 43

S1. Reagents and General Equipment

All reagents were purchased from commercial sources (Alfa Aesar, Apollo Scientific Ltd., Fisher Scientific UK Ltd., Fluorochem UK Ltd., TCI UK Ltd. and Sigma–Aldrich Company Ltd) unless stated otherwise, and used without further purification.

Dry solvents were obtained by means of a MBBRAUN MB SPS-800™ purification system.

Flash column chromatography was performed using Geduran® Si60 (40-63 μM, Merck, Germany) as the stationary phase and thin layer chromatography was performed on pre-coated silica gel-plates (0.25 mm thick, $60F_{254}$, Merck, Germany) and observed under UV light irradiation.

^{1}H, ^{13}C, ^{19}F NMR spectroscopic data was acquired using either a Bruker Avance (500 MHz), a Bruker Avance III (500 MHz) or a Bruker Avance II (400 MHz) spectrometer, at a constant temperature of 25 ºC unless stated otherwise. ^{1}H and ^{13}C chemical shifts are reported in parts per million (ppm) from high to low field and referenced to the literature values for chemical shift of the residual non-deuterated solvent, with respect to tetramethylsilane. ^{19}F NMR chemical shifts are referenced to $CFCl_3$ (0.00 ppm).

All melting points were measured using a Stuart SMP30 melting point apparatus.

Mass spectra were recorded on a Micromass GCT spectrometer for chemical ionisation (CI) using isobutene as the ionising gas. Electron spray ionisation (ESI) spectra were performed on a Micromass LCT spectrometer operating in positive or negative mode, *m/z* values are reported in Daltons.

S2. Synthetic Procedures

1,3-di-tert-butyl-5-(iodoethynyl)benzene,[1] 4-(3,5-di-tert-butylphenyl)-5-iodo-1-(perfluorophenyl)-1H-1,2,3-triazole[1] (**ITr**) and tris-(benzyltriazolylmethyl)amine[2] (**TBTA**) were prepared accordingly to previously published procedures.

Synthesis of 1-(4-(4-(3,5-di-tert-butylphenyl)-5-iodo-1*H*-1,2,3-triazol-1-yl)-2,3,5,6-tetrafluoro phenyl)pyridin-4(1*H*)-one (1)

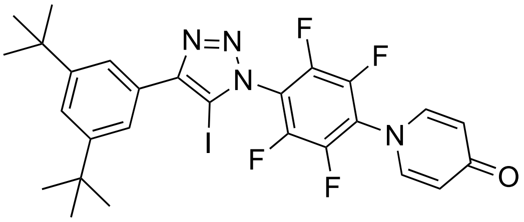

Potassium carbonate (0.25 g, 1.8 mmol) was added to a solution of 4-hydroxypyridine (0.17 g, 1.8 mmol) in DMF (7.0 mL), and the mixture was stirred at room temperature for 40 minutes before adding iodotriazole **ITr** (1.00 g, 1.8 mmol). The resulting suspension was stirred at room temperature overnight. The solution was extracted with ethyl acetate and the organic extracts were combined and washed with aqueous LiCl saturated solution, dried over $MgSO_4$, filtered and concentrated *in vacuo*. Purification of the product was achieved by column chromatography of the crude (1:1 to 9:1 petroleum ether: EtOAc 9:1) yielding product **1** as a yellow solid (1.17 g, 1.5 mmol, 80%). Crystals suitable for single crystal X-ray diffraction were obtained upon cooling of a saturated toluene solution.

M.p. > 139 ºC dec.. $^{1}H\{^{19}F\}$ NMR (500.1 MHz; $CDCl_3$): δ 7.86 (d, J = 1.6 Hz, 2H), 7.52 (m, 1H), 7.50 – 7.48 (m, 2H), 6.53 – 6.52 (m, 2H), 1.39 (s, 18H). ^{19}F NMR (470.6 MHz; $CDCl_3$): δ −139.72–−139.79 (m, 2F), −145.64–−145.71 (m, 2F). ^{13}C NMR (125.7 MHz; $CDCl_3$): δ 178.5, 151.7, 151.4, 144.8–143.2 (m), 142.7–141.2 (m), 139.7, 127.9, 124.2 (t, J = 12.8 Hz), 123.4, 121.9, 119.8, 117.2 (t, J = 12.8 Hz), 79.9, 35.2, 31.5. HRMS pESI (*m/z*) $[M+Na]^+$ calcd. for $C_{27}H_{25}OF_4IN_4Na$ 647.0901; found 647.0890.

Synthesis of phenol-appended iodotriazole 2-H

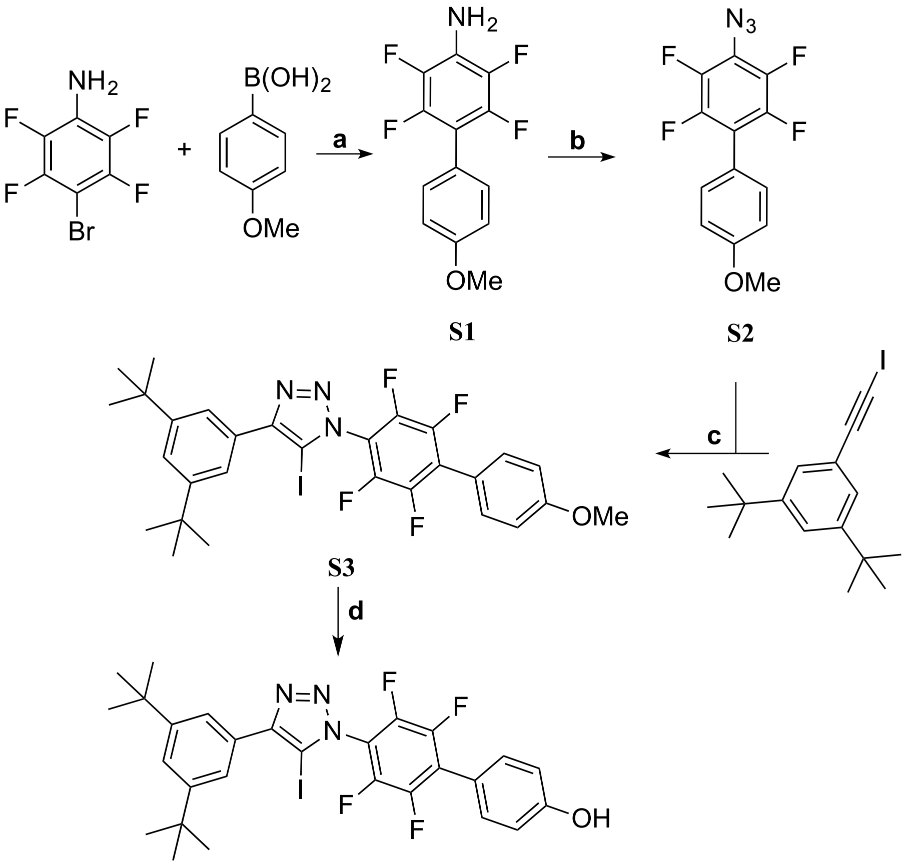

Scheme S1. Synthesis of phenol-appended iodotriazole **2**-H. Reagents and conditions: (a) $Pd(PPh_3)_4$, *aq.* 2 M K_2CO_3, 6.8:1 toluene:ethanol, 90 ºC, 24 hs; (b) $NaNO_2$, **TFA**, 0 ºC, 2 hs; (c) **TBTA**, CuI, THF, RT, 12 hs; (d) 1 M BBr_3 in DCM, −78 ºC, 1 h.

2,3,5,6-tetrafluoro-4'-methoxy-[1,1'-biphenyl]-4-amine (**S1**). 4-bromo-2,3,5,6-tetrafluroaniline (2.44 g, 10.0 mmol), (4-methoxyphenol)boronic acid (1.52 g, 10.0 mmol) and an aqueous potassium carbonate solution (2 M, 10.5 mL) were dissolved in toluene (34.0 mL) and ethanol (5.0 mL). $Pd(PPh_3)_4$ (1.03 g, 0.89 mmol) was added under an argon atmosphere and the reaction mixture was heated at 90 ºC for 24 hours. After this time, the crude mixture was concentrated under reduced pressure, then redissolved in ethyl acetate and washed with brine. The organic phase was then dried over $MgSO_4$ and concentrated again under reduced pressure. The resulting crude was purified by flash chromatography, (SiO_2, 1:1 to 4:6 petroleum ether: DCM) to afford aniline **S1** as a white needle-like solid (1.81 g, 6.67 mmol, 68%).

M.p. 113.2−114.6 ºC. ^{1}H NMR (400.1 MHz, $CDCl_3$): δ 7.37 − 7.34 (m, 2H), 7.00 − 6.97 (m, 2H), 4.00 (s, 2H), 3.85 (s, 3H). ^{19}F NMR (470.5 MHz, $CDCl_3$): δ −146.86 − −146.96 (m, 2F), −162.28 − −162.38 (m, 2F). ^{13}C NMR (125.7 MHz, $CDCl_3$): δ 159.6, 145.3–143.2 (m, 1C), 138.0–135.9

(m, 1C), 131.6 (t, $J = 1.6$ Hz, 1C), 125.1–124.9 (m, 1C), 120.2 (t, $J = 2.0$ Hz, 1C), 114.1, 108.2 (m, $J = 17.3$ Hz, 1C), 55.4.
HRMS ESI– (*m/z*) $[M-H]^-$ calcd. for $C_{13}H_8F_4NO$, 270.0548; found 270.0546.

4-azido-2,3,5,6-tetrafluoro-4'-methoxy-1,1'-biphenyl (**S2**). Aniline **S1** (1.00 g, 3.68 mmol), was dissolved in trifluoroacetic acid (9.0 mL). $NaNO_2$ (0.26 g, 4.42 mmol) was slowly added at 0 ºC and the resulting mixture was stirred for 1 hour at this temperature. NaN_3 (0.36 g, 5.52 mmol) was then added over a period of 10 minutes and the reaction stirred for a further hour at 0 ºC. Ethyl ether was added to quench the reaction and the crude mixture washed with an aqueous $NaHCO_3$ saturated solution and brine. The organic layer was dried over $MgSO_4$ and concentrated under reduced pressure to afford azide **S2** as a white solid (0.96 g, 3.09 mmol, 84%).
M.p. > 110 ºC dec.. ^{1}H NMR (500.1 MHz, d_6-DMSO): δ 7.44 – 7.42 (m, 2H), 7.11 – 7.09 (m, 2H), 3.82 (s, 3H). ^{19}F NMR (470.5 MHz, d_6-DMSO): δ −144.85 – −144.92 (m, 2F), −152.58 – −152.65 (m, 2F). ^{13}C NMR (125.7 MHz, d_6-DMSO): δ 160.0, 144.5 – 142.4 (m, 1C), 141.5 – 139.4 (m, 1C), 131.4, 118.3 – 118.1 (m, 1C), 118.0, 115.3 (t, $J = 17.4$ Hz, 1C), 114.4, 55.3.
HRMS CI+ (*m/z*) $[M+H]^+$ calcd. for $C_{13}H_8F_4N_3O$, 298.0603; found 298.0676.

4-(3,5-di-*tert*-butylphenyl)-5-iodo-1-(2,3,5,6-tetrafluoro-4'-methoxy-[1,1'-biphenyl]-4-yl)-1*H*-1,2,3-triazole (**S3**). **TBTA** (0.30 g, 0.57 mmol) was stirred in dry THF (25.0 mL) with CuI (0.11 g, 0.57 mmol) for 20 minutes. 1,3-Di-*tert*-butyl-5-(iodoethynyl)benzene (1.94 g, 5.69 mmol) and azide **S2** (1.69 g, 5.69 mmol) were dissolved in THF (10.0 mL) and added in a single portion to the catalyst mixture. After stirring the reaction mixture for 5 hours at room temperature, this was quenched by adding an aqueous ammonium hydroxide solution (10%, 20.1 mL) and concentrated under reduced pressure. The crude mix was redissolved in ether and washed with water and brine, then dried over $MgSO_4$ filtered and concentrated *in vacuo*. The crude mixture was purified *via* column chromatography (SiO_2, 4:1 petroleum ether:DCM to straight DCM gradient) to afford iodotriazole **S3** as a white powder (2.5 g, 3.92 mmol, 69%). Crystals suitable for single crystal X-ray diffraction were obtained upon cooling of a saturated toluene solution.
M.p. 113–114.6 ºC. ^{1}H NMR (500.1 MHz, $CDCl_3$): δ 7.91 (d, $J = 1.8$ Hz, 2H), 7.52–7.50 (m, 3H), 7.09 – 7.02 (m, 2H), 3.90 (s, 3H), 1.41 (s, 18H). ^{19}F NMR (470.6 MHz, $CDCl_3$): δ −141.86 – −141.99 (m, 2F), −144.11 – −144.22 (m, 2F). ^{13}C NMR (125.7 MHz, $CDCl_3$): δ 160.9, 151.4, 151.3, 145.1–143.1 (m, 1C), 144.5–142.3 (m, 1C), 131.7, 128.4, 124.0 (t, $J = 16.4$ Hz, 1C), 123.3, 122.0, 118.3, 114.8–114.6 (m, 1C), 114.5, 80.0, 55.6, 35.3, 31.6.
HRMS ESI+ (*m/z*) $[M+Na]^+$ calcd. for $C_{29}H_{28}F_4IN_3NaO$, 660.1111; found 660.1097.

4'-(4-(3,5-di-*tert*-butylphenyl)-5-iodo-1*H*-1,2,3-triazol-1-yl)-2',3',5',6'-tetrafluoro-[1,1'-biphenyl]-4-ol (**2**-H). Iodotriazole **S3** (0.49 g, 0.78 mmol) was dissolved in DCM (3.5 mL) and cooled to −78 ºC under an Ar atmosphere. Boron tribromide (3.1 mL, 1 M DCM solution) was added slowly at this temperature and the reaction mixture was allowed to warm to room temperature then stirred for 1 hour. After this time, the reaction was quenched by slow addition of cold aqueous saturated $NaHCO_3$ solution, transferred into a separating funnel and extracted with DCM. The organic layers were dried with $MgSO_4$, filtered and concentrated *in vacuo* to afford **2**-H as pale brown crystals (0.39 g, 0.64 mmol, 82%). Crystals suitable for single crystal X-ray diffraction were obtained upon cooling of a saturated THF solution.

M.p. > 124 ºC dec.. ^{1}H NMR (500.1 MHz, $CDCl_3$): δ 7.90 (d, *J* = 1.8 Hz, 2H), 7.52 (t, *J* = 1.8 Hz, 1H), 7.46–7.44 (m, 2H), 7.04–7.02 (m, 2H), 5.85 (br s, 1H), 1.41 (s, 18H). ^{19}F NMR (470.6 MHz, $CDCl_3$): δ −143.31 – −143.50 (m, 2F), −147.42 – −147.59 (m, 2F). ^{13}C NMR (125.7 MHz, $CDCl_3$): δ 157.5, 151.5, 151.4, 145.9–142.3 (m, 1C), 145.2–141.5 (m, 1C), 131.9 (t, *J* = 2.2 Hz, 1C), 128.2, 124.2 (t, *J* = 16.4 Hz, 1C), 123.4, 122.1, 118.1, 116.1, 114.8–114.3 (m, 1C), 80.3, 35.3, 31.6.

HRMS ESI+ (*m/z*) $[M+Na]^+$ calcd. for $C_{28}H_{26}F_4IN_3NaO$, 646.0949; found 646.0921.

S3. Solid state structures of 1 and solution state studies

As described in the main text, the same crystal growth method, *i.e.* slow evaporation from a saturated toluene solution, afforded two different batches of crystals. The acquisition of X-ray diffraction data on both crystalline materials allowed the unravelling the polymorphic character of pyridone-appended iodotriazole **1**. Although the poorer quality of the second batch of crystal did not permit structure solution to an R-factor lower than 15.4%, the data was of sufficient quality to identify the presence of a homodimeric assembly within the solid state structure (**Figure S1**).

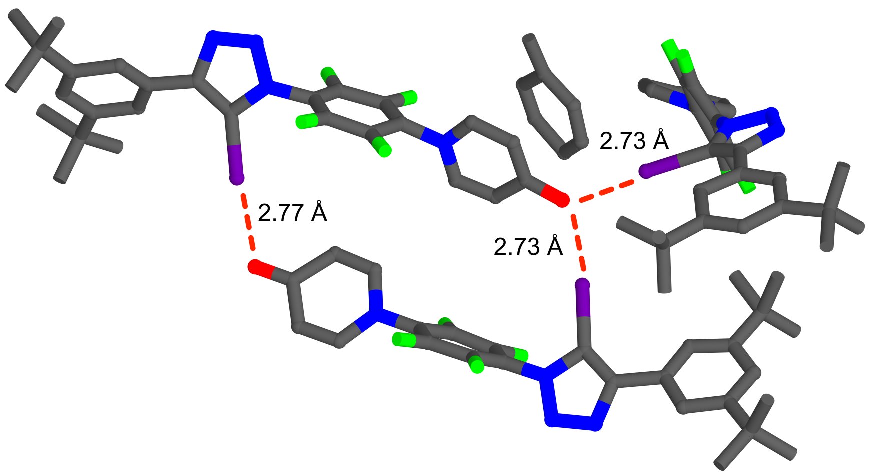

Figure S1. Solid state structure, determined from X-ray diffraction data, of the polymorph of pyridone-appended iodotriazole **1**. The homodimeric assembly is characterised by two XB interactions 2.77 and 2.73 Å long; a third XB contact (2.73 Å) involves the second lone pair of the carbonyl oxygen atom of **1** with the iodine atom of a third iodotriazole unit.

Interestingly, the XB interactions within the homodimeric assembly slightly differ in length as a result of the bifurcated halogen bonding interaction involving only one of the two carbonyl oxygen atoms. Further attempts to crystallise this polymorph of **1** from toluene afforded only the crystalline phase reported in the main text.

The stability of dimer [**1•1**] in d_8-toluene solution was determined[3] by fitting ^1H chemical shift changes to a dimerisation model using the WinEQNMR[4] program. Data for this fitting process were generated by recording the 500.1 $^1H\{^{19}F\}$ NMR spectra of independently prepared solutions of **1** in d_8-toluene in the concentration range 80 to 1 mM. The best fit of the appropriate dimerisation model to the $^1H\{^{19}F\}$ NMR data afforded a K_a of 2.3 ± 0.3 M^{-1} as reported in the main text.

S4. Crystal structures of iodotriazoles S3 and 2-H

Given the highly crystalline character of intermediates **S3** and **2**-H, the crystal packing of these iodotriazole scaffolds was examined to provide a preliminary evaluation of the halogen bonding ability of this particular iodotriazole design. **Figure S2** shows the crystal structure of the methyl protected phenol appended iodotriazole **S3**.

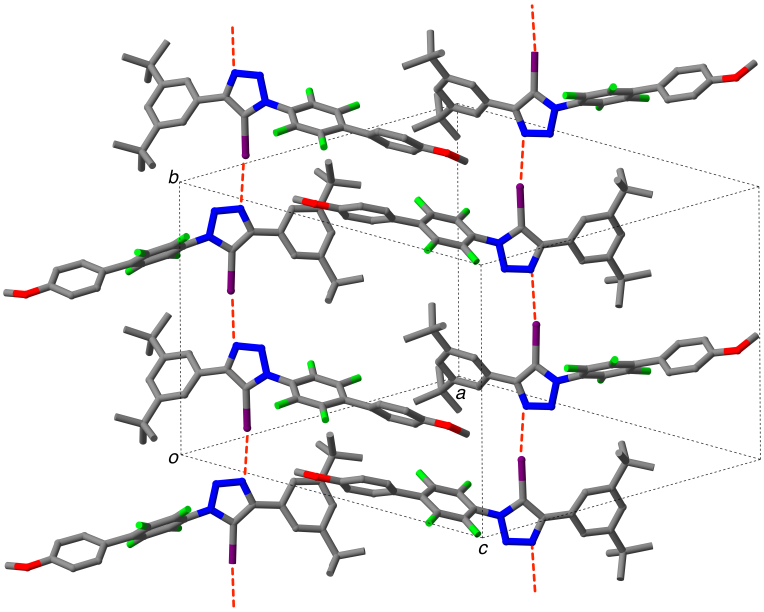

Figure S2. Solid state structure, determined by X-ray diffraction data, of iodotriazole **S3**. The presence of the methyl group prevents the oxygen atom behaving as a XB acceptor. The XB formed occurs between the iodine atom and the nitrogen atom at the 3- position the iodotriazole ring (*d*(N•••I)=2.818 Å, ∠(C–I•••N)=171.3º).

Similarly to previously synthesised unsubstituted iodotriazoles,[1] the observed crystal packing is the result of the XB contact between the iodine atom of one iodotriazole unit with the nitrogen atom in the 3- position of the consecutive iodotriazole thus creating antiparallel halogen bonded tapes. Evidently, the presence of the methyl group hinders the ability of the oxygen atom to perform as a XB acceptor. The replacement of the methyl group with a smaller hydrogen atom, as in **2**-H, provided both a XB acceptor and a HB donor in the form of a phenol group. Despite the reported solid-state evidence of phenol groups able to engage in both noncovalent interaction at the same time reported by Desiraju[5,6] and Aakeröy,[7] the sole short contact observed in the crystal structure of **2**-H is the HB between the phenolic proton and the ethereal oxygen atom of a THF molecule with whom **2**-H cocrystallises (**Figure S3a**). The resulting crystal packing is obtained by this interaction solely, no other noncovalent interactions were observed (**Figure S3b**).

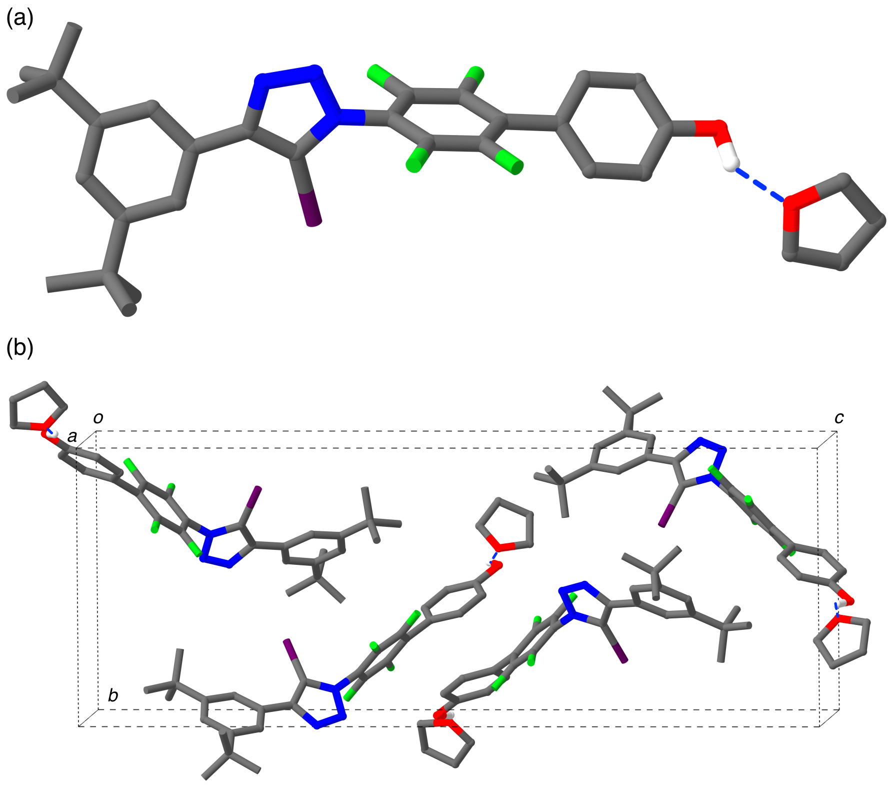

Figure S3. (a) Solid state structure, determined from X-ray diffraction data, of a single **2**-H molecule hydrogen bonding to a THF molecule (d(O–H•••O)=1.797 Å). (b) Crystal packing of the **2**-H in the unit cell showing that no bulk XB-based arrangement is achieved.

Seemingly, the HB donating character of the phenol group for this particular molecular design overpowers the XB donating properties of the iodotriazole.

S5. Deprotonation of phenol 2-H and related control experiments

In a typical experiment, 12 mg (0.02 mmol) phenol-appended iodotriazole **2**-H were dissolved in 0.98 mL of CD_3CN spiked with 4-fluoronitrobenzene (5 mM, δ_F referenced at −104.0 ppm). This solution was treated with 0.02 mL of a 1 M methanol solution of tetrabutylammonium hydroxide (**TBAOH**) thus affording a 20 mM concentration of phenolate **2-TBA** (**Figure S4a**). High-resolution mass spectrometry analysis (HRMS) of this solution by electrospray ionisation in positive and negative mode confirmed the presence of the **2-TBA** salt (HRMS ESI– (*m/z*) $[\mathbf{2}–H]^-$ calcd. for $C_{28}H_{25}F_4IN_3O$, 622.0984; found 622.0983 and HRMS ESI+ (*m/z*) $[\mathbf{TBA}]^+$ calcd. for $C_{16}H_{36}N$, 242.2848; found 242.2834).

A 0.6 mL aliquot of the 20 mM CD_3CN solution of **2-TBA** was transferred in an NMR tube and a 470.6 MHz ^{19}F NMR spectrum was recorded. The dilution data shown in **Figure S4c** were generated by progressive dilutions from 20 mM down to 0.5 mM in 6 steps. The concentration dependence of the ^{19}F resonances of the **2-TBA** solutions were interpreted as the result of the dissociation of the doubly halogen-bonded $[\mathbf{2•2}]^{2–}$ dimer and fitted[4] to a 1:1 binding isotherm. The best fit of the appropriate dimerisation model to the ^{19}F NMR data afforded a K_a of 510 ± 30 M^{-1} as reported in the main text, proving that, at 20 mM concentration of $\mathbf{2}^-$, most of this compound in solution is in its dimeric form.

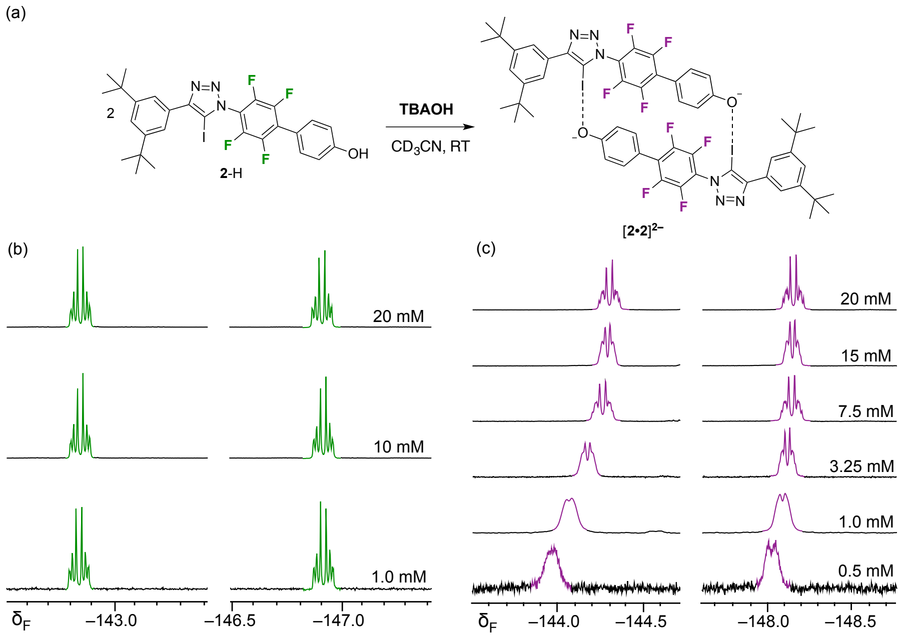

Figure S4. (a) Quantitative deprotonation of **2**-H by treatment with one equivalent of **TBAOH**. (b) Partial 470.6 MHz ^{19}F NMR spectra of **2**-H at different dilutions showing that in this range of concentration in CD_3CN the ^{19}F resonances of **2**-H are not concentration dependent. (c) Partial 470.6 MHz ^{19}F NMR spectra of $[\mathbf{2•2}]^{2–}$ showing the effect of dilution on the halogen-bonded dimer.

As a control experiment, a 20 mM solution of **2**-H was subjected to a similar dilution procedure. **Figure S4b** shows the lack of ^{19}F NMR chemical shift changes in **2**-H upon dilution, thus confirming the inability of the phenol-appended iodotriazole **2**-H to partake in any HB- or XB-based assembly. A second control experiment was conducted by monitoring the deprotonation of pentafluorophenol (**Figure S5a**) and its subsequent dilution of the pentafluorophenoxide solution in the 20 to 1 mM concentration range at 298 K in CD_3CN by 376.4 MHz ^{19}F NMR spectroscopy (**Figure S5b**).

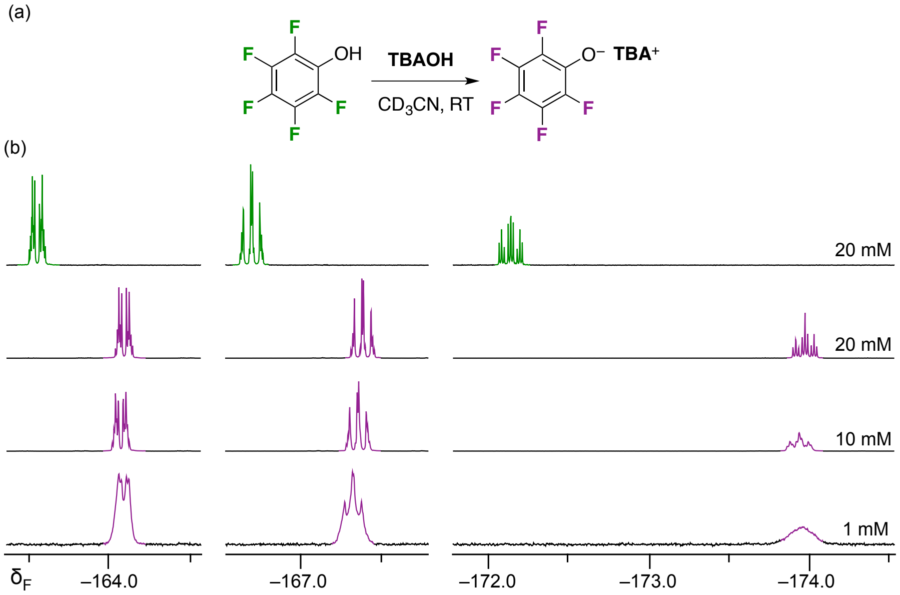

Figure S5. (a) Deprotonation of pentafluorophenol (20 mM in CD_3CN) with **TBAOH** (1 M in methanol). (b) Partial 376.4 MHz ^{19}F NMR spectra of a 20 mM solution of pentafluorophenol and pentafluorophenoxide **TBA** salt showing that the chemical shift relative to the ^{19}F resonances of the phenoxide salt are not concentration dependent.

Once again, the minimal changes in the ^{19}F NMR spectrum at the three concentrations shown in **Figure S5b**, namely 20 mM, 10 mM and 1 mM, show that there is little to no dependence between the NMR resonances of a simple phenoxide salt and its concentration in a polar aprotic solvent such as CD_3CN.

S6. Single point XB association constant determination

Iodotriazole **S4**[1] was chosen as the model XB donor to measure the single point association constant with a phenoxide anion in order to establish the effective molarity of homodimer [**2•2**]$^{2-}$. In first instance, a fresh solution of tetra-*n*-butylammonium phenolate was titrated in a 10 mM CD_3CN solution of **S4**. Unfortunately this measurement was hampered by the tendency of iodotriazole **S4** to undergo reductive deiodination in presence of the **TBA** phenolate salt to afford triazole **S4**-H. **Figure S7** shows the NMR characterisation of the isolated prototriazole **S4**-H.

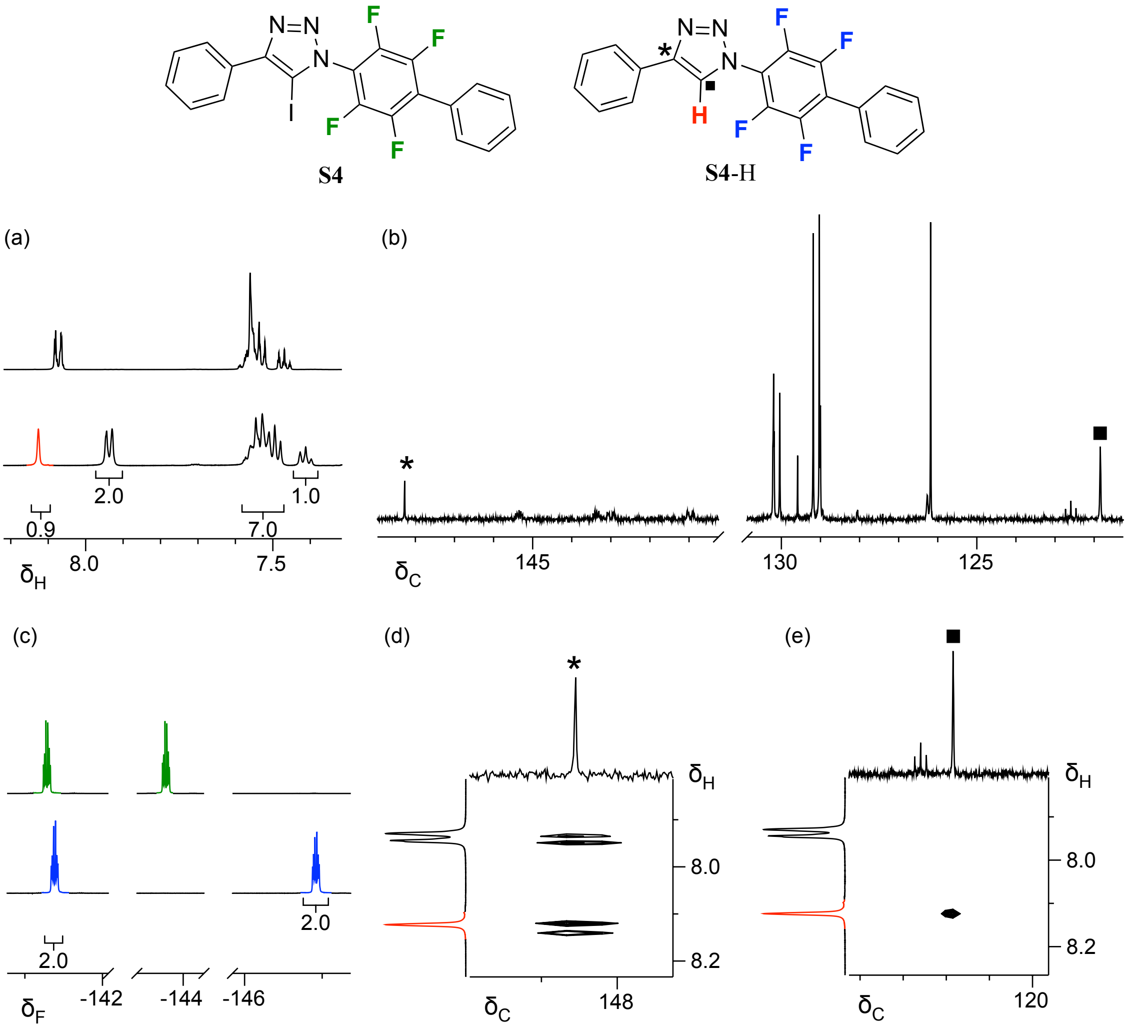

Figure S7. NMR characterisation of 5*H*-triazole **S4**-H in $CDCl_3$ at RT: (a) stack of partial 500.1 MHz ^{1}H NMR spectra of iodotriazole **S4** (top) and prototriazole **S4**-H (bottom); (b) partial 125.7 MHz ^{13}C NMR spectrum of **S4**-H; (c) stack of partial 470.5 MHz ^{19}F NMR spectra of iodotriazole **S4** (top) and prototriazole **S4**-H (bottom); (d) partial ^{1}H–^{13}C HMBC NMR spectrum of **S4**-H; (e) partial ^{1}H–^{13}C HSQC NMR spectrum of **S4**-H.

Such reactivity was not observed when the titration was performed with **DBU**H phenolate as the XB acceptor. This allowed us to titrate a 10 mM solution of **S4** with 1 to 10 equivalents of **DBU**H phenolate in presence of 4-fluoro-toluene as internal standard (10 mM, δ_F referenced at –120 ppm).

Fitting[4] of the ^{19}F chemical shift changes of **S4** to a 1:1 binding model using the WinEQNMR program afforded an association constant of 6.6 ± 0.2 M^{-1}.

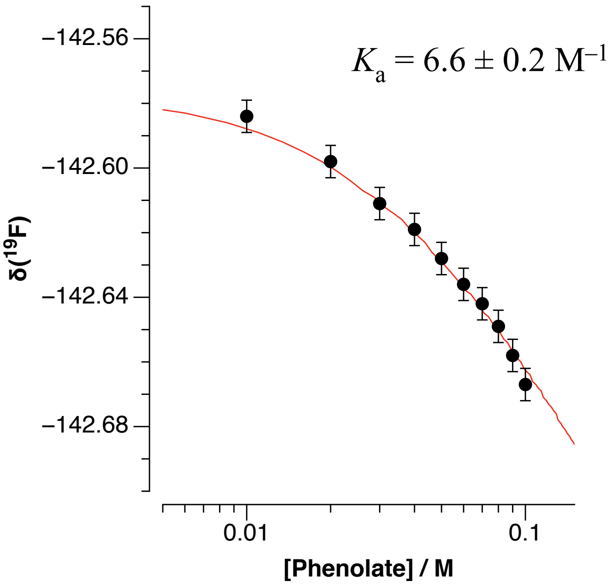

Figure S8. Variation of the ^{19}F chemical shift of iodotriazole **S4** as a function of the concentration of **DBU**H phenolate added. The solid red line represents the best fit of a dimerisation model to the data.

S7. Preparation of [2•2]•(DBUH)$_2$

A 500 mM solution of iodotriazole **2**-H was prepared in 0.6 mL of CD_3CN and increasing amounts of **DBU** were added sequentially (**Figure S6a**) to this solution. The extent of deprotonation of **2**-H was monitored by 376.4 MHz ^{19}F NMR spectroscopy. Upon addition of 1 to 7 equivalents of base, small upfield chemical shift changes occurred, along with the progressive yellow colouring of the solution, indicating a progressive shifting of the equilibrium towards the anionic form of **2** (**Figure S6b**). Ultimately, the addition of 15 equivalents of **DBU** caused the precipitation of **[2•2]•(DBU**H)$_2$ as confirmed by the single crystal X-ray diffraction analysis (**Figure S6c**).

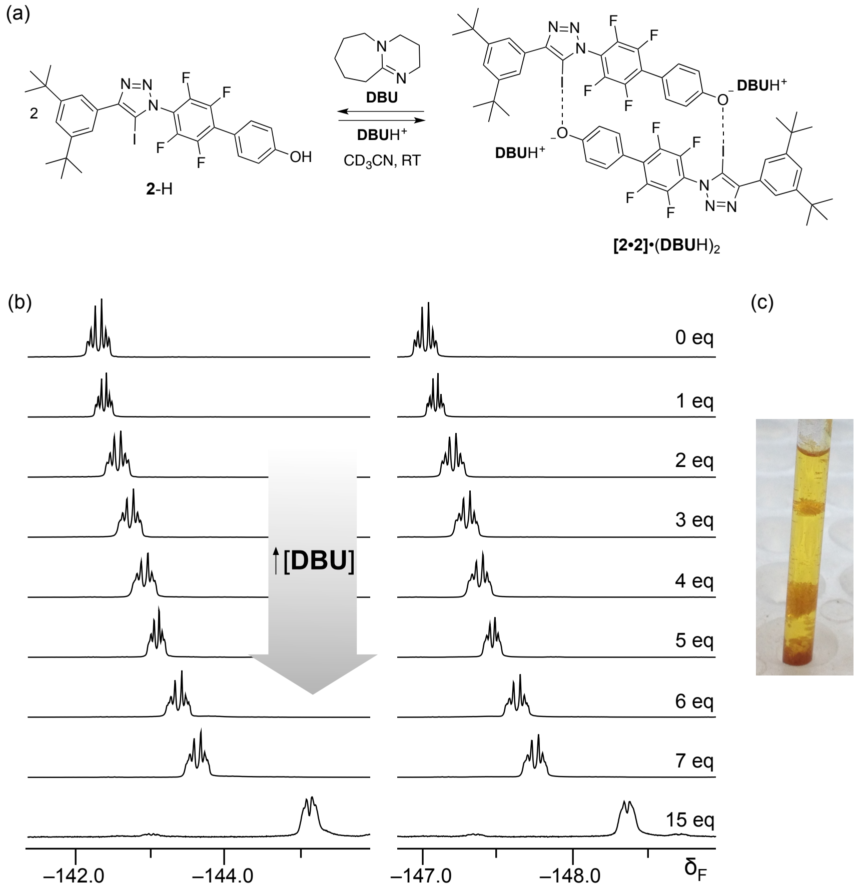

Figure S6. (a) DBU mediated phenol/phenolate equilibrium between **2**-H and **[2•2]•(DBU**H)$_2$ in CD_3CN at 298 K (b) Partial 376.4 MHz ^{19}F NMR of a 500 mM solution of **2**-H in CD_3CN at 298 K with increasing amounts of **DBU** (c) Picture showing the precipitation of crystals of **[2•2]•(DBU**H)$_2$ after addition of 15 equivalents of **DBU**.

High-resolution mass spectrometry analysis (HRMS) of the filtered solid material by electrospray ionisation in positive and negative mode confirmed the identity of the **2**-**DBU**H salt (HRMS ESI– (*m/z*) [**2**–H]$^-$ calcd. for $C_{28}H_{25}F_4IN_3O$, 622.0984; found 622.0985 and HRMS ESI+ (*m/z*) [**DBU**H]$^+$ calcd. for $C_{16}H_{36}N$, 153.1392; found 153.1377).

S8. DFT calculations

All calculations were performed using Gaussian09[8] suite of programs – revision D.01 was used in all calculations. Calculation were performed using TPSSh[9] functional and the def2-TZVP basis set[10] of Weigend and Ahlrichs. This basis set is not standard in Gaussian09 and was introduced using the `GenECP` keyword using an appropriately-formatted input block for the basis set generated from data obtained from the Basis Set Exchange[11] (https://bse.pnl.gov/bse/portal). An effective core potential on iodine,[12] which replaces 28 valence electrons on each iodine atom in the structure was used in all calculations. The basis set is all-electron for all other elements.

All geometries were optimized fully in internal (keyword: `opt`) or cartesian (keyword: `opt=cartesian`) coordinates using the default optimisation protocols within Gaussian09. Stationary points were characterised by means of a vibrational analysis (keyword: `freq`) and zero-point energy corrections and other thermodynamic parameters, used in the calculation of interactions energies, were derived[13] from this analysis. Population analyses using the Natural Bond Orbital (NBO) method were performed using the NBO6 program[14,15] in a two-stage procedure. The input for NBO6 was generated using Gaussian09 (keyword: `population=nboread`) and the .47 file generated by this calculation was then edited and processed using the `gennbo` script provided with the NBO6 distribution. All analyses used the 8-byte integer version of NBO6 dated March 2015 compiled from source using *gfortran* (Version 4.4.7).

The basis set superposition error was calculated using the counterpoise method[16] as implemented within Gaussian09 (keyword: `counterpoise = 2`).

Halogen bonds were visualized using the NCIPLOT[17] program. SCF densities written as an extended wavefunction file from Gaussian09 (rev. D.01) and NCIPLOT used to generate Gaussian cube files from which isosurfaces could be visualised using VMD[18] with s = 0.5 and a colours (blue (attractive) to red (repulsive)) mapped on to $-0.05 < \rho < 0.05$.

S9. References

[1] L. Maugeri, J. Asencio-Hernández, T. Lebl, D. B. Cordes, A. Slawin, M.-A. Delsuc, D. Philp, *Chem. Sci.* **2016**, DOI 10.1039/C6SC01974A.

[2] T. R. Chan, R. Hilgraf, K. B. Sharpless, V. V. Fokin, *Org. Lett.* **2004**, *6*, 2853–2855.

[3] P. Thordarson, *Chem. Soc. Rev.* **2011**, *40*, 1305–1323.

[4] M. J. Hynes, *J. Chem. Soc. Dalt. Trans.* **1993**, 311–312.

[5] A. Mukherjee, S. Tothadi, G. R. Desiraju, *Acc. Chem. Res.* **2014**, *47*, 2514–2524.

[6] A. Mukherjee, G. R. Desiraju, *IUCrJ* **2014**, *1*, 49–60.

[7] C. B. Aakeröy, S. Panikkattu, P. D. Chopade, J. Desper, *CrystEngComm* **2013**, *15*, 3125–3136.

[8] Gaussian 09, Revision A.02 or D.01, M. J. Frisch, G. W. Trucks, H. B. Schlegel, G. E. Scuseria, M. A. Robb, J. R. Cheeseman, G. Scalmani, V. Barone, B. Mennucci, G. A. Petersson, H. Nakatsuji, M. Caricato, X. Li, H. P. Hratchian, A. F. Izmaylov, J. Bloino, G. Zheng, J. L. Sonnenberg, M. Hada, M. Ehara, K. Toyota, R. Fukuda, J. Hasegawa, M. Ishida, T. Nakajima, Y. Honda, O. Kitao, H. Nakai, T. Vreven, J. A. Montgomery, Jr., J. E. Peralta, F. Ogliaro, M. Bearpark, J. J. Heyd, E. Brothers, K. N. Kudin, V. N. Staroverov, R. Kobayashi, J. Normand, K. Raghavachari, A. Rendell, J. C. Burant, S. S. Iyengar, J. Tomasi, M. Cossi, N. Rega, J. M. Millam, M. Klene, J. E. Knox, J. B. Cross, V. Bakken, C. Adamo, J. Jaramillo, R. Gomperts, R. E. Stratmann, O. Yazyev, A. J. Austin, R. Cammi, C. Pomelli, J. W. Ochterski, R. L. Martin, K. Morokuma, V. G. Zakrzewski, G. A. Voth, P. Salvador, J. J. Dannenberg, S. Dapprich, A. D. Daniels, Ö. Farkas, J. B. Foresman, J. V. Ortiz, J. Cioslowski, and D. J. Fox, Gaussian, Inc., Wallingford CT, 2009.

[9] J. Tao, J. P. Perdew, V. N. Staroverov, G. E. Scuseria, *Phys. Rev. Lett.* **2003**, *91*, 146401.

[10] F. Weigend, R. Ahlrichs, *Phys. Chem. Chem. Phys.* **2005**, *7*, 3297–3305.

[11] K. L. Schuchardt, B. T. Didier, T. Elsethagen, L. Sun, V. Gurumoorthi, J. Chase, J. Li, T. L. Windus, *J. Chem. Inf. Model.* **2007**, *47*, 1045–1052.

[12] K. A. Peterson, *J. Chem. Phys.* **2003**, *119*, 11113–11123.

[13] J. W. Ochterski, Thermochemistry in Gaussian; Gaussian Inc.: Wallingford, CT, 2000.

[14] E. D. Glendening, C. R. Landis, F. Weinhold, *J. Comput. Chem.* **2013**, *34*, 1429–1437.

[15] E. D. Glendening, J, K. Badenhoop, A. E. Reed, J. E. Carpenter, J. A. Bohmann, C. M. Morales, C. R. Landis, and F. Weinhold, Theoretical Chemistry Institute, University of Wisconsin, Madison (2013).

[16] S. F. Boys, F. Bernardi, *Mol. Phys.* **1970**, *19*, 553–566.

[17] E. R. Johnson, S. Keinan, P. Mori Sánchez, J. Contreras García, A. J. Cohen, W. Yang, *J. Am. Chem. Soc.* **2010**, *132*, 6498–6506.

[18] W. Humphrey, A. Dalke and K. Schulten, *J. Molec. Graphics,* **1996**, *14*, 33-38. http://www.ks.uiuc.edu/Research/vmd/

S10. Crystallographic Informations

The CCDC numbers for compounds **1**, **S3**, **2**-H and [**2•2**]•(**DBU**H)$_2$ are 1488032-1488035.

1-(4-(4-(3,5-di-tert-butylphenyl)-5-iodo-1*H*-1,2,3-triazol-1-yl)-2,3,5,6-tetrafluoro phenyl)pyridin-4(1*H*)-one (**1**)

Data Collection

A colorless prism crystal of $C_{34}H_{33}F_4IN_4O$ having approximate dimensions of 0.200 × 0.200 × 0.200 mm was mounted in a loop. All measurements were made on a Rigaku XtaLAB P200 diffractometer using multi-layer mirror monochromated Mo-Kα radiation.

Cell constants and an orientation matrix for data collection corresponded to a primitive monoclinic cell with dimensions:

a = 12.469(3) Å
b = 17.723(4) Å   β = 112.991(4)º
c = 15.596(4) Å
V = 3172.8(13) Å^3

For Z = 4 and F.W. = 716.56, the calculated density is 1.500 g/cm^3. The reflection conditions of:

h0l: h+l = 2n
0k0: k = 2n

uniquely determine the space group to be:

P2$_1$/n (#14)

The data were collected at a temperature of −180 ± 1ºC to a maximum 2θ value of 50.7º.

Data Reduction

Of the 36305 reflections were collected, where 5647 were unique (R_{int} = 0.1242); equivalent reflections were merged. Data were collected and processed using CrystalClear (Rigaku).[1]

The linear absorption coefficient, μ, for Mo-Kα radiation is 10.667 cm^{-1}. An empirical absorption correction was applied which resulted in transmission factors ranging from 0.416 to 0.808. The data were corrected for Lorentz and polarization effects.

Structure Solution and Refinement

The structure was solved by heavy-atom Patterson methods[2] and expanded using Fourier techniques. The non-hydrogen atoms were refined anisotropically. Hydrogen atoms were refined using the riding model. The final cycle of full-matrix least-squares refinement[3] on F^2 was based on 5647 observed reflections and 404 variable parameters and converged (largest parameter shift was 0.00 times its esd) with unweighted and weighted agreement factors of:

$$R1 = \sum ||Fo| - |Fc|| / \sum |Fo| = 0.0507$$

$$wR2 = [\sum (w (Fo^2 - Fc^2)^2)/ \sum w(Fo^2)^2]^{1/2} = 0.1254$$

The goodness of fit[4] was 0.95. Unit weights were used. The maximum and minimum peaks on the final difference Fourier map corresponded to 3.25 and −1.32 e[illegible]$Å^3$, respectively.

Neutral atom scattering factors were taken from International Tables for Crystallography (IT), Vol. C, Table 6.1.1.4.[5] Anomalous dispersion effects were included in Fcalc;[6] the values for Δf' and Δf" were those of Creagh and McAuley.[7] The values for the mass attenuation coefficients are those of Creagh and Hubbell.[8] All calculations were performed using the CrystalStructure[9] crystallographic software package except for refinement, which was performed using SHELXL2013.[10]

References

(1) CrystalClear: Data Collection and Processing Software, Rigaku Corporation (1998-2014). Tokyo 196-8666, Japan.

(2) PATTY: Beurskens, P.T., Admiraal, G., Behm, H., Beurskens, G., Smits, J.M.M. and Smykalla, C. (1991). Z. f. Kristallogr. Suppl.4, p.99.

(3) Least Squares function minimized: (SHELXL2013)

$\sum w(F_o^2 - F_c^2)^2$ where w = Least Squares weights.

(4) Goodness of fit is defined as:

$[\sum w(F_o^2 - F_c^2)^2/(N_o - N_v)]^{1/2}$

where: N_O = number of observations
N_V = number of variables

(5) International Tables for Crystallography, Vol.C (1992). Ed. A.J.C. Wilson, Kluwer Academic Publishers, Dordrecht, Netherlands, Table 6.1.1.4, pp. 572.

(6) Ibers, J. A. & Hamilton, W. C.; Acta Crystallogr., 17, 781 (1964).

(7) Creagh, D. C. & McAuley, W.J .; "International Tables for Crystallography", Vol C, (A.J.C. Wilson, ed.), Kluwer Academic Publishers, Boston, Table 4.2.6.8, pages 219-222 (1992).

(8) Creagh, D. C. & Hubbell, J.H..; "International Tables for Crystallography", Vol C, (A.J.C. Wilson, ed.), Kluwer Academic Publishers, Boston, Table 4.2.4.3, pages 200-206 (1992).

(9) CrystalStructure 4.1: Crystal Structure Analysis Package, Rigaku Corporation (2000-2014). Tokyo 196-8666, Japan.

(10) SHELXL2013: Sheldrick, G. M. (2008). Acta Cryst. A64, 112-122.

EXPERIMENTAL DETAILS

A. Crystal Data

Empirical Formula	$C_{34}H_{33}F_4IN_4O$
Formula Weight	716.56
Crystal Color, Habit	colorless, prism
Crystal Dimensions	0.200 × 0.200 × 0.200 mm
Crystal System	monoclinic
Lattice Type	Primitive
Lattice Parameters	a = 12.469(3) Å<br>b = 17.723(4) Å<br>c = 15.596(4) Å<br>β = 112.991(4)º<br>V = 3172.8(13) $Å^3$
Space Group	$P2_1/n$ (#14)
Z value	4
D_{calc}	1.500 g/cm^3
F_{000}	1448.00
μ(Mo-Kα)	10.667 cm^{-1}

B. Intensity Measurements

Diffractometer	XtaLAB P200
Radiation	Mo-Kα (λ = 0.71075 Å)<br>multi-layer mirror monochromated
Voltage, Current	45kV, 66mA
Temperature	−180.0 ºC
Detector Aperture	83.8 × 70.0 mm
Pixel Size	0.172 mm
$2\theta_{max}$	50.7º
No. of Reflections Measured Total:	36305<br>Unique: 5647 (R_{int} = 0.1242)
Corrections	Lorentz-polarization<br>Absorption<br>(trans. factors: 0.416 – 0.808)

C. Structure Solution and Refinement

Structure Solution	Patterson Methods (DIRDIF99 PATTY)
Refinement	Full-matrix least-squares on F^2
Function Minimized	$\sum w(F_o^2 - F_c^2)^2$
Least Squares Weights	$w = 1/ [\sigma^2(F_o^2) + (0.0848 \cdot P)^2 + 0.0000 \cdot P]$ where $P = (Max(F_o^2,0) + 2 F_c^2)/3$
$2\theta_{max}$ cutoff	50.7°
Anomalous Dispersion	All non-hydrogen atoms
No. Observations (All reflections)	5647
No. Variables	404
Reflection/Parameter Ratio	13.98
Residuals: R1 (I>2.00σ(I))	0.0507
Residuals: R (All reflections)	0.0589
Residuals: wR2 (All reflections)	0.1254
Goodness of Fit Indicator	0.954
Max Shift/Error in Final Cycle	0.000
Maximum peak in Final Diff. Map	3.25 $e^-/Å^3$
Minimum peak in Final Diff. Map	−1.32 $e^-/Å^3$

4-(3,5-di-*tert*-butylphenyl)-5-iodo-1-(2,3,5,6-tetrafluoro-4'-methoxy-[1,1'-biphenyl]-4-yl)-1*H*-1,2,3-triazole (S3)

Data Collection

A colourless prism crystal of $C_{29}H_{28}F_4IN_3O$ having approximate dimensions of 0.100 × 0.030 × 0.030 mm was mounted in a loop. All measurements were made on a Rigaku Saturn724 diffractometer using graphite monochromated Mo-Kα radiation.

Cell constants and an orientation matrix for data collection corresponded to a primitive monoclinic cell with dimensions:

a = 25.213(7) Å
b = 13.978(3) Å β = 112.879(4)º
c = 27.637(7) Å
V = 8974(4) $Å^3$

For Z = 12 and F.W. = 637.46, the calculated density is 1.415 g/cm^3. The reflection conditions of:

h0l: h+l = 2n
0k0: k = 2n

uniquely determine the space group to be:

$P2_1/n$ (#14)

The data were collected at a temperature of −148 ±1 ºC to a maximum 2θ value of 50.8º.

Data Reduction

Of the 109125 reflections were collected, where 16446 were unique (R_{int} = 0.2103); equivalent reflections were merged. Data were collected and processed using CrystalClear (Rigaku).[1]

The linear absorption coefficient, μ, for Mo-Kα radiation is 11.207 cm^{-1}. An empirical absorption correction was applied which resulted in transmission factors ranging from 0.789 to 0.967. The data were corrected for Lorentz and polarization effects.

Structure Solution and Refinement

The structure was solved by heavy-atom Patterson methods[2] and expanded using Fourier techniques. The non-hydrogen atoms were refined anisotropically. Hydrogen atoms were refined using the riding model. The final cycle of full-matrix least-squares refinement[3] on F^2 was based on 16446 observed reflections and 1027 variable parameters and converged (largest parameter shift was 0.04 times its esd) with unweighted and weighted agreement factors of:

$$R1 = \sum ||Fo| - |Fc|| / \sum |Fo| = 0.1077$$

$$wR2 = [\sum (w (Fo^2 - Fc^2)^2)/ \sum w(Fo^2)^2]^{1/2} = 0.3145$$

The goodness of fit[4] was 1.04. Unit weights were used. The maximum and minimum peaks on the final difference Fourier map corresponded to 3.73 and −1.27 e[illegible]/Å^{3}, respectively.

Neutral atom scattering factors were taken from International Tables for Crystallography (IT), Vol. C, Table 6.1.1.4.[5] Anomalous dispersion effects were included in Fcalc;[6] the values for Δf' and Δf" were those of Creagh and McAuley.[7] The values for the mass attenuation coefficients are those of Creagh and Hubbell.[8] All calculations were performed using the CrystalStructure[9] crystallographic software package except for refinement, which was performed using SHELXL Version 2014/7.[10]

References

(1) CrystalClear: Data Collection and Processing Software, Rigaku Corporation (1998-2015). Tokyo 196-8666, Japan.

(2) PATTY: Beurskens, P.T., Admiraal, G., Behm, H., Beurskens, G., Smits, J.M.M. and Smykalla, C. (1991). Z. f. Kristallogr. Suppl.4, p.99.

(3) Least Squares function minimized: (SHELXL2013)

$\sum w(F_o^2 - F_c^2)^2$ where w = Least Squares weights.

(4) Goodness of fit is defined as:

$[\sum w(F_o^2 - F_c^2)^2/(N_o - N_v)]^{1/2}$

where: N_O = number of observations
N_V = number of variables

(5) International Tables for Crystallography, Vol.C (1992). Ed. A.J.C. Wilson, Kluwer Academic Publishers, Dordrecht, Netherlands, Table 6.1.1.4, pp. 572.

(6) Ibers, J. A. & Hamilton, W. C.; Acta Crystallogr., 17, 781 (1964).

(7) Creagh, D. C. & McAuley, W.J .; "International Tables for Crystallography", Vol C, (A.J.C. Wilson, ed.), Kluwer Academic Publishers, Boston, Table 4.2.6.8, pages 219-222 (1992).

(8) Creagh, D. C. & Hubbell, J.H..; "International Tables for Crystallography", Vol C, (A.J.C. Wilson, ed.), Kluwer Academic Publishers, Boston, Table 4.2.4.3, pages 200-206 (1992).

(9) CrystalStructure 4.2: Crystal Structure Analysis Package, Rigaku Corporation (2000-2015). Tokyo 196-8666, Japan.

(10) SHELXL Version 2014/7: Sheldrick, G. M. (2008). Acta Cryst. A64, 112-122.

EXPERIMENTAL DETAILS

A. Crystal Data

Empirical Formula	$C_{29}H_{28}F_4IN_3O$
Formula Weight	637.46
Crystal Color, Habit	colourless, prism
Crystal Dimensions	0.100 × 0.030 × 0.030 mm
Crystal System	monoclinic
Lattice Type	Primitive
Lattice Parameters	a = 25.213(7) Å<br>b = 13.978(3) Å<br>c = 27.637(7) Å<br>β = 112.879(4)º<br>V = 8974(4) $Å^3$
Space Group	$P2_1/n$ (#14)
Z value	12
D_{calc}	1.415 g/cm^3
F_{000}	3840.00
μ(Mo-Kα)	11.207 cm^{-1}

B. Intensity Measurements

Diffractometer	Saturn724
Radiation	MoKα (λ = 0.71075 Å)<br>graphite monochromated
Voltage, Current	50 kV, 20 mA
Temperature	−148.0 ºC
Detector Aperture	72.8 × 72.8 mm
Pixel Size	0.070 mm
$2\theta_{max}$	50.8º
No. of Reflections Measured	Total: 109125<br>Unique: 16446 (R_{int} = 0.2103)
Corrections	Lorentz-polarization<br>Absorption<br>(trans. factors: 0.789–0.967)

C. Structure Solution and Refinement

Structure Solution	Patterson Methods (DIRDIF99 PATTY)
Refinement	Full-matrix least-squares on F^2
Function Minimized	$\sum w(F_o^2 - F_c^2)^2$
Least Squares Weights	$w = 1/[\sigma^2(F_o^2) + (0.1362 \cdot P)^2 + 110.0794 \cdot P]$<br>where $P = (Max((F_o^2,0) + 2(F_c^2)/3$
$2\theta_{max}$ cutoff	50.8º
Anomalous Dispersion	All non-hydrogen atoms
No. Observations (All reflections)	16446
No. Variables	1027
Reflection/Parameter Ratio	16.01
Residuals: R1 ($I>2.00\sigma(I)$)	0.1077
Residuals: R (All reflections)	0.1938
Residuals: wR2 (All reflections)	0.3145
Goodness of Fit Indicator	1.043
Max Shift/Error in Final Cycle	0.038
Maximum peak in Final Diff. Map	$3.73\ e^{-}/\text{Å}^3$
Minimum peak in Final Diff. Map	$-1.27\ e^{-}/\text{Å}^3$

4'-(4-(3,5-di-*tert*-butylphenyl)-5-iodo-1*H*-1,2,3-triazol-1-yl)-2',3',5',6'-tetrafluoro-[1,1'-biphenyl]-4-ol (**2**-H).

Data Collection

A colorless prism crystal of $C_{32}H_{34}F_4IN_3O_2$ having approximate dimensions of 0.240 × 0.080 × 0.070 mm was mounted in a loop. All measurements were made on a Rigaku XtaLAB P200 diffractometer using multi-layer mirror monochromated Mo-Kα radiation.

The crystal-to-detector distance was 45.06 mm.

Cell constants and an orientation matrix for data collection corresponded to a primitive orthorhombic cell with dimensions:

a = 8.4431(6) Å
b = 11.7892(12) Å
c = 31.416(3) Å
V = 3127.1(5) $Å^3$

For Z = 4 and F.W. = 695.54, the calculated density is 1.477 g/cm^3. The reflection conditions of:

h00: h = 2n
0k0: k = 2n
00l: l = 2n

uniquely determine the space group to be:

$P2_12_12_1$ (#19)

The data were collected at a temperature of −100 ± 1 ºC to a maximum 2θ value of 50.7º. A total of 1080 oscillation images were collected. A sweep of data was done using ω scans from −100.0 to 80.0º in 0.50º step, at χ=45.0º and Φ= 0.0º. The exposure rate was 10.0 [sec./º]. The detector swing angle was −10.38º. A second sweep was performed using ω scans from −100.0 to 80.0º in 0.50º step, at χ=45.0º and Φ = 90.0º. The exposure rate was 10.0 [sec./º]. The detector swing angle was -

10.38º. Another sweep was performed using ω scans from −100.0 to 80.0º in 0.50º step, at χ=45.0º and Φ= 180.0º. The exposure rate was 10.0 [sec./º]. The detector swing angle was −10.38º. The crystal-to-detector distance was 45.06 mm. Readout was performed in the 0.172 mm pixel mode.

Data Reduction

Of the 38083 reflections were collected, where 5690 were unique (R_{int} = 0.0269); equivalent reflections were merged. Data were collected and processed using CrystalClear (Rigaku).[1]

The linear absorption coefficient, μ, for Mo-Kα radiation is 10.811 cm^{-1}. An empirical absorption correction was applied which resulted in transmission factors ranging from 0.853 to 0.927. The data were corrected for Lorentz and polarization effects.

Structure Solution and Refinement

The structure was solved by heavy-atom Patterson methods[2] and expanded using Fourier techniques. The non-hydrogen atoms were refined anisotropically. Some hydrogen atoms were refined isotropically, some were refined using the riding model, and the rest were included in fixed positions. The final cycle of full-matrix least-squares refinement[3] on F^2 was based on 5690 observed reflections and 388 variable parameters and converged (largest parameter shift was 0.00 times its esd) with unweighted and weighted agreement factors of:

$$R1 = \sum ||Fo| - |Fc|| / \sum |Fo| = 0.0245$$

$$wR2 = [\sum (w (Fo^2 - Fc^2)^2)/ \sum w(Fo^2)^2]^{1/2} = 0.0634$$

The goodness of fit[4] was 1.05. Unit weights were used. The maximum and minimum peaks on the final difference Fourier map corresponded to 0.54 and −0.33 e[illegible]/Å^3, respectively. The final Flack parameter[5] was −0.024(4), indicating that the value is out of statistically acceptable range.[6] It is recommended to repeat least-squares refinement carefully until getting meaningful value, or, in the worst case, to average Friedel pairs.

Neutral atom scattering factors were taken from International Tables for Crystallography (IT), Vol. C, Table 6.1.1.4.[7] Anomalous dispersion effects were included in Fcalc;[8] the values for Δf' and Δf" were those of Creagh and McAuley.[9] The values for the mass attenuation coefficients are those of Creagh and Hubbell.[10] All calculations were performed using the CrystalStructure[11] crystallographic software package except for refinement, which was performed using SHELXL2013.[12]

References

(1) CrystalClear: Data Collection and Processing Software, Rigaku Corporation (1998-2014). Tokyo 196-8666, Japan.

(2) PATTY: Beurskens, P.T., Admiraal, G., Behm, H., Beurskens, G., Smits, J.M.M. and Smykalla, C. (1991). Z. f. Kristallogr. Suppl.4, p.99.

(3) Least Squares function minimized: (SHELXL2013)

$$\sum w(F_o^2 - F_c^2)^2$$ where w = Least Squares weights.

(4) Goodness of fit is defined as:

$$[\sum w(F_o^2 - F_c^2)^2/(N_o - N_v)]^{1/2}$$

where: N_O = number of observations
N_V = number of variables

(5) Parsons, S. and Flack, H. (2004), Acta Cryst. A60, s61.

(6) Flack, H.D. and Bernardinelli (2000), J. Appl. Cryst. 33, 114-1148.

(7) International Tables for Crystallography, Vol.C (1992). Ed. A.J.C. Wilson, Kluwer Academic Publishers, Dordrecht, Netherlands, Table 6.1.1.4, pp. 572.

(8) Ibers, J. A. & Hamilton, W. C.; Acta Crystallogr., 17, 781 (1964).

(9) Creagh, D. C. & McAuley, W.J .; "International Tables for Crystallography", Vol C, (A.J.C. Wilson, ed.), Kluwer Academic Publishers, Boston, Table 4.2.6.8, pages 219-222 (1992).

(10) Creagh, D. C. & Hubbell, J.H..; "International Tables for Crystallography", Vol C, (A.J.C. Wilson, ed.), Kluwer Academic Publishers, Boston, Table 4.2.4.3, pages 200-206 (1992).

(11) CrystalStructure 4.1: Crystal Structure Analysis Package, Rigaku Corporation (2000-2014). Tokyo 196-8666, Japan.

(12) SHELXL2013: Sheldrick, G. M. (2008). Acta Cryst. A64, 112-122.

EXPERIMENTAL DETAILS

A. Crystal Data

Empirical Formula	$C_{32}H_{34}F_4IN_3O_2$
Formula Weight	695.54
Crystal Color, Habit	colorless, prism
Crystal Dimensions	0.240 × 0.080 × 0.070 mm
Crystal System	orthorhombic
Lattice Type	Primitive
Lattice Parameters	a = 8.4431(6) Å<br>b = 11.7892(12) Å<br>c = 31.416(3) Å<br>V = 3127.1(5) $Å^3$
Space Group	$P2_12_12_1$ (#19)
Z value	4
D_{calc}	1.477 g/cm^3
F_{000}	1408.00
μ(MoKα)	10.811 cm^{-1}

B. Intensity Measurements

Diffractometer	XtaLAB P200
Radiation	MoKα (λ = 0.71075 Å)<br>multi-layer mirror monochromated
Voltage, Current	45 kV, 66 mA
Temperature	−100.0 ºC
Detector Aperture	83.8 × 70.0 mm
Data Images	1080 exposures
ω oscillation Range (χ =45.0, Φ = 0.0)	−100.0–80.0º
Exposure Rate	10.0 sec./º
Detector Swing Angle	−10.38º
ω oscillation Range (χ =45.0, Φ = 90.0)	−100.0–80.0º
Exposure Rate	10.0 sec./º
Detector Swing Angle	−10.38º
ω oscillation Range (χ =45.0, Φ = 180.0)	−100.0–80.0º
Exposure Rate	10.0 sec./º
Detector Swing Angle	−10.38º
Detector Position	45.06 mm
Pixel Size	0.172 mm
$2\theta_{max}$	50.7º
No. of Reflections Measured	Total: 38083<br>Unique: 5690 (R_{int} = 0.0269)<br>Parsons quotients (Flack x parameter):<br>2263
Corrections	Lorentz-polarization<br>Absorption<br>(trans. factors: 0.853 – 0.927)

C. Structure Solution and Refinement

Structure Solution	Patterson Methods (DIRDIF99 PATTY)
Refinement	Full-matrix least-squares on F^2
Function Minimized	$\sum w(F_o^2 - F_c^2)^2$
Least Squares Weights	$w = 1/ [\sigma^2(F_o^2) + (0.0370 \cdot P)^2 + 1.5440 \cdot P]$<br>where $P = (Max((F_o^2,0) + 2(F_c^2)/3$
$2\theta_{max}$ cutoff	50.7°
Anomalous Dispersion	All non-hydrogen atoms
No. Observations (All reflections)	5690
No. Variables	388
Reflection/Parameter Ratio	14.66
Residuals: R1 (I>2.00σ(I))	0.0245
Residuals: R (All reflections)	0.0265
Residuals: wR2 (All reflections)	0.0634
Goodness of Fit Indicator	1.046
Flack parameter (Parsons' quotients = 2263)	−0.024(4)
Max Shift/Error in Final Cycle	0.001
Maximum peak in Final Diff. Map	0.54 e□/Å^3
Minimum peak in Final Diff. Map	−0.33 e□/ Å^3

2,3,4,5,7,8,9,10-octahydropyrido[1,2-*a*][1,3]diazepin-1-ium 4'-(4-(3,5-di-*tert*-butylphenyl)-5-iodo-1*H*-1,2,3-triazol-1-yl)-2',3',5',6'-tetrafluoro-[1,1'-biphenyl]-4-olate ([**2•2**]•**DBU**H_2)

Data Collection

A yellow prism crystal of $C_{37}H_{42}F_4IN_5O$ having approximate dimensions of 0.210 × 0.120 × 0.120 mm was mounted in a loop. All measurements were made on a Rigaku XtaLAB P200 diffractometer using multi-layer mirror monochromated Mo-Kα radiation.

The crystal-to-detector distance was 44.97 mm.

Cell constants and an orientation matrix for data collection corresponded to an I-centered monoclinic cell with dimensions:

a = 20.258(3) Å
b = 13.9884(13) Å   β = 103.187(10)º
c = 26.734(4) Å
V = 7376.1(17) $Å^3$

For Z = 8 and F.W. = 775.67, the calculated density is 1.397 g/cm^3. Based on the reflection conditions of:

hkl: h+k+l = 2n
h0l: h = 2n

packing considerations, a statistical analysis of intensity distribution, and the successful solution and refinement of the structure, the space group was determined to be:

I2/a (#15)

The data were collected at a temperature of −100 ± 1ºC to a maximum 2θ value of 50.8º. A total of 1080 oscillation images were collected. A sweep of data was done using ω scans from −100.0 to 80.0º in 0.50º step, at χ=45.0º and Φ = 0.0º. The exposure rate was 10.0 [sec./º]. The detector swing angle was −10.37º. A second sweep was performed using ω scans from −100.0 to 80.0º in 0.50º

step, at χ=45.0° and Φ = 90.0°. The exposure rate was 10.0 [sec./°]. The detector swing angle was −10.37°. Another sweep was performed using ω scans from −100.0 to 80.0° in 0.50° step, at χ=45.0° and Φ = 180.0°. The exposure rate was 10.0 [sec./°]. The detector swing angle was −10.37°. The crystal-to-detector distance was 44.97 mm. Readout was performed in the 0.172 mm pixel mode.

Data Reduction

Of the 44203 reflections were collected, where 6762 were unique (R_{int} = 0.0604); equivalent reflections were merged. Data were collected and processed using CrystalClear (Rigaku).[1]

The linear absorption coefficient, μ, for Mo-Kα radiation is 9.242 cm^{-1}. An empirical absorption correction was applied which resulted in transmission factors ranging from 0.770 to 0.895. The data were corrected for Lorentz and polarization effects.

Structure Solution and Refinement

The structure was solved by heavy-atom Patterson methods[2] and expanded using Fourier techniques. The non-hydrogen atoms were refined anisotropically. Some hydrogen atoms were refined isotropically, some were refined using the riding model, and the rest were included in fixed positions. The final cycle of full-matrix least-squares refinement[3] on F^2 was based on 6762 observed reflections and 473 variable parameters and converged (largest parameter shift was 0.00 times its esd) with unweighted and weighted agreement factors of:

$$R1 = \sum ||Fo| - |Fc|| / \sum |Fo| = 0.0387$$

$$wR2 = [\sum (w (Fo^2 - Fc^2)^2)/ \sum w(Fo^2)^2]^{1/2} = 0.0882$$

The goodness of fit[4] was 1.03. Unit weights were used. The maximum and minimum peaks on the final difference Fourier map corresponded to 0.80 and −0.43 $e^{-}/Å^3$, respectively.

Neutral atom scattering factors were taken from International Tables for Crystallography (IT), Vol. C, Table 6.1.1.4.[5] Anomalous dispersion effects were included in Fcalc;[6] the values for Δf' and Δf" were those of Creagh and McAuley.[7] The values for the mass attenuation coefficients are those of

Creagh and Hubbell.[8] All calculations were performed using the CrystalStructure[9] crystallographic software package except for refinement, which was performed using SHELXL2013.[10]

References

(1) CrystalClear: Data Collection and Processing Software, Rigaku Corporation (1998-2014). Tokyo 196-8666, Japan.

(2) PATTY: Beurskens, P.T., Admiraal, G., Behm, H., Beurskens, G., Smits, J.M.M. and Smykalla, C. (1991). Z. f. Kristallogr. Suppl.4, p.99.

(3) Least Squares function minimized: (SHELXL2013)

$$\sum w(F_o^2 - F_c^2)^2 \quad \text{where } w = \text{Least Squares weights.}$$

(4) Goodness of fit is defined as:

$$[\sum w(F_o^2 - F_c^2)^2/(N_o - N_v)]^{1/2}$$

where: N_O = number of observations
N_V = number of variables

(5) International Tables for Crystallography, Vol.C (1992). Ed. A.J.C. Wilson, Kluwer Academic Publishers, Dordrecht, Netherlands, Table 6.1.1.4, pp. 572.

(6) Ibers, J. A. & Hamilton, W. C.; Acta Crystallogr., 17, 781 (1964).

(7) Creagh, D. C. & McAuley, W.J .; "International Tables for Crystallography", Vol C, (A.J.C. Wilson, ed.), Kluwer Academic Publishers, Boston, Table 4.2.6.8, pages 219-222 (1992).

(8) Creagh, D. C. & Hubbell, J.H..; "International Tables for Crystallography", Vol C, (A.J.C. Wilson, ed.), Kluwer Academic Publishers, Boston, Table 4.2.4.3, pages 200-206 (1992).

(9) CrystalStructure 4.1: Crystal Structure Analysis Package, Rigaku Corporation (2000-2014). Tokyo 196-8666, Japan.

(10) SHELXL2013: Sheldrick, G. M. (2008). Acta Cryst. A64, 112-122.

EXPERIMENTAL DETAILS

A. Crystal Data

Empirical Formula	$C_{37}H_{42}F_4IN_5O$
Formula Weight	775.67
Crystal Color, Habit	yellow, prism
Crystal Dimensions	0.210 × 0.120 × 0.120 mm
Crystal System	monoclinic
Lattice Type	I-centered
Lattice Parameters	a = 20.258(3) Å<br>b = 13.9884(13) Å<br>c = 26.734(4) Å<br>β = 103.187(10)º<br>V = 7376.1(17) $Å^3$
Space Group	I2/a (#15)
Z value	8
D_{calc}	1.397 g/cm^3
F_{000}	3168.00
μ(MoKα)	9.242 cm^{-3}

B. Intensity Measurements

Diffractometer	XtaLAB P200
Radiation	MoKα (λ = 0.71075 Å)<br>multi-layer mirror monochromated
Voltage, Current	45 kV, 66 mA
Temperature	−100.0 ºC
Detector Aperture	83.8 × 70.0 mm
Data Images	1080 exposures
ω oscillation Range (χ=45.0, Φ=90.0)	−100.0–80.0º
Exposure Rate	10.0 sec./º
Detector Swing Angle	−10.37º
ω oscillation Range (χ=45.0, Φ=0.0)	−100.0–80.0º
Exposure Rate	10.0 sec./º
Detector Swing Angle	−10.37º
ω oscillation Range (χ=45.0, Φ=180.0)	−100.0–80.0º
Exposure Rate	10.0 sec./º
Detector Swing Angle	−10.37º
Detector Position	44.97 mm
Pixel Size	0.172 mm
$2\theta_{max}$	50.8º
No. of Reflections Measured	Total: 44203<br>Unique: 6762 (R_{int} = 0.0604)
Corrections	Lorentz-polarization<br>Absorption<br>(trans. factors: 0.770–0.895)

C. Structure Solution and Refinement

Structure Solution	Patterson Methods (DIRDIF99 PATTY)
Refinement	Full-matrix least-squares on F^2
Function Minimized	$\sum w(F_o^2 - F_c^2)^2$
Least Squares Weights	$w = 1/ [\sigma^2(F_o^2) + (0.0370 \cdot P)^2 + 1.5440 \cdot P]$<br>where $P = (Max((F_o^2,0) + 2(F_c^2)/3$
$2\theta_{max}$ cutoff	50.8°
Anomalous Dispersion	All non-hydrogen atoms
No. Observations (All reflections)	6762
No. Variables	473
Reflection/Parameter Ratio	14.30
Residuals: R1 ($I>2.00\sigma$ (I))	0.0387
Residuals: R (All reflections)	0.0695
Residuals: wR2 (All reflections)	0.0882
Goodness of Fit Indicator	1.028
Max Shift/Error in Final Cycle	0.000
Maximum peak in Final Diff. Map	0.80 $e^-/Å^3$
Minimum peak in Final Diff. Map	−0.43 $e^-/Å^3$

S11. NMR Spectra

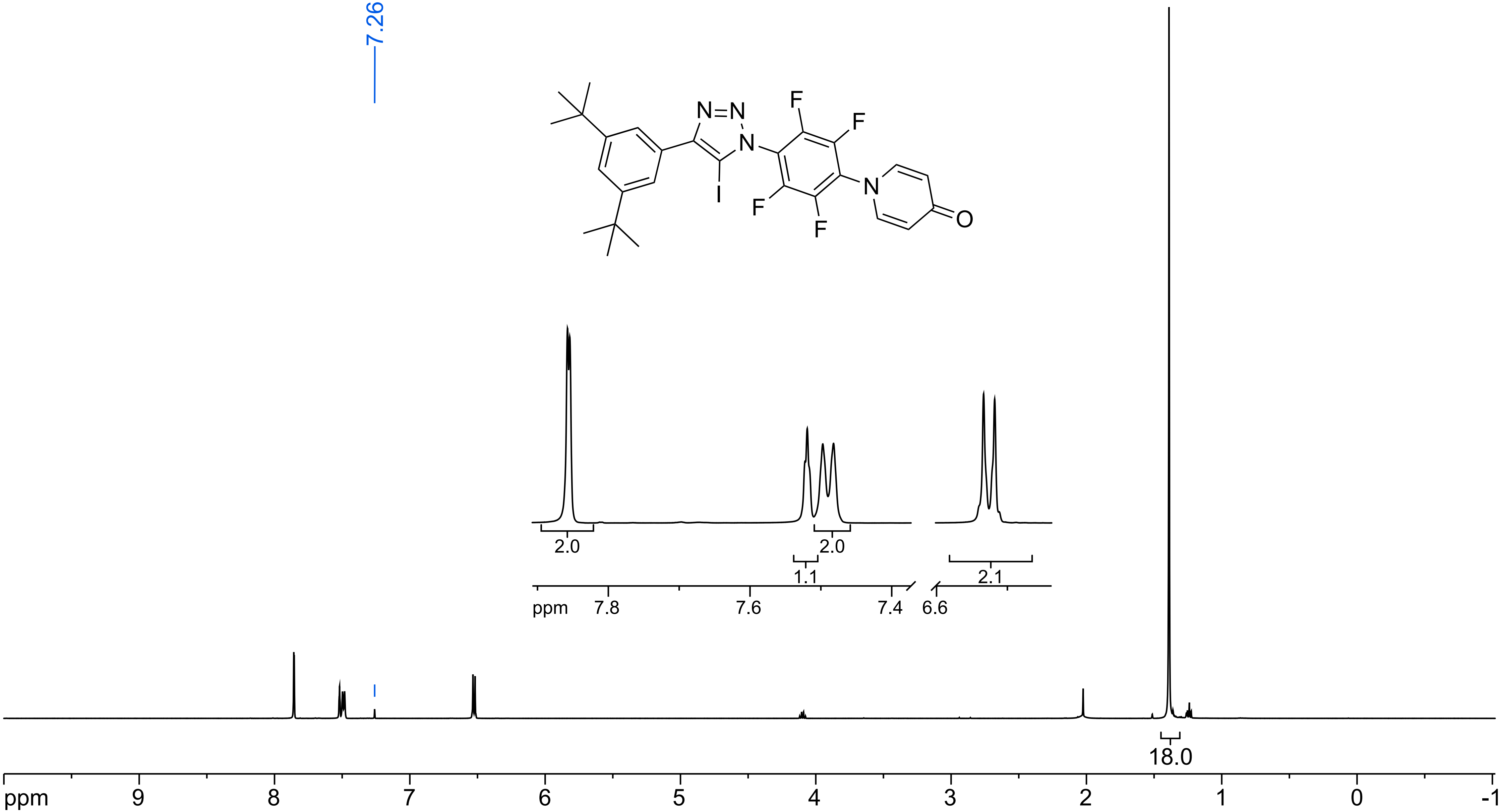

^{1}H NMR (500.1 MHz, RT, $CDCl_3$) spectrum of iodotriazole **1**.

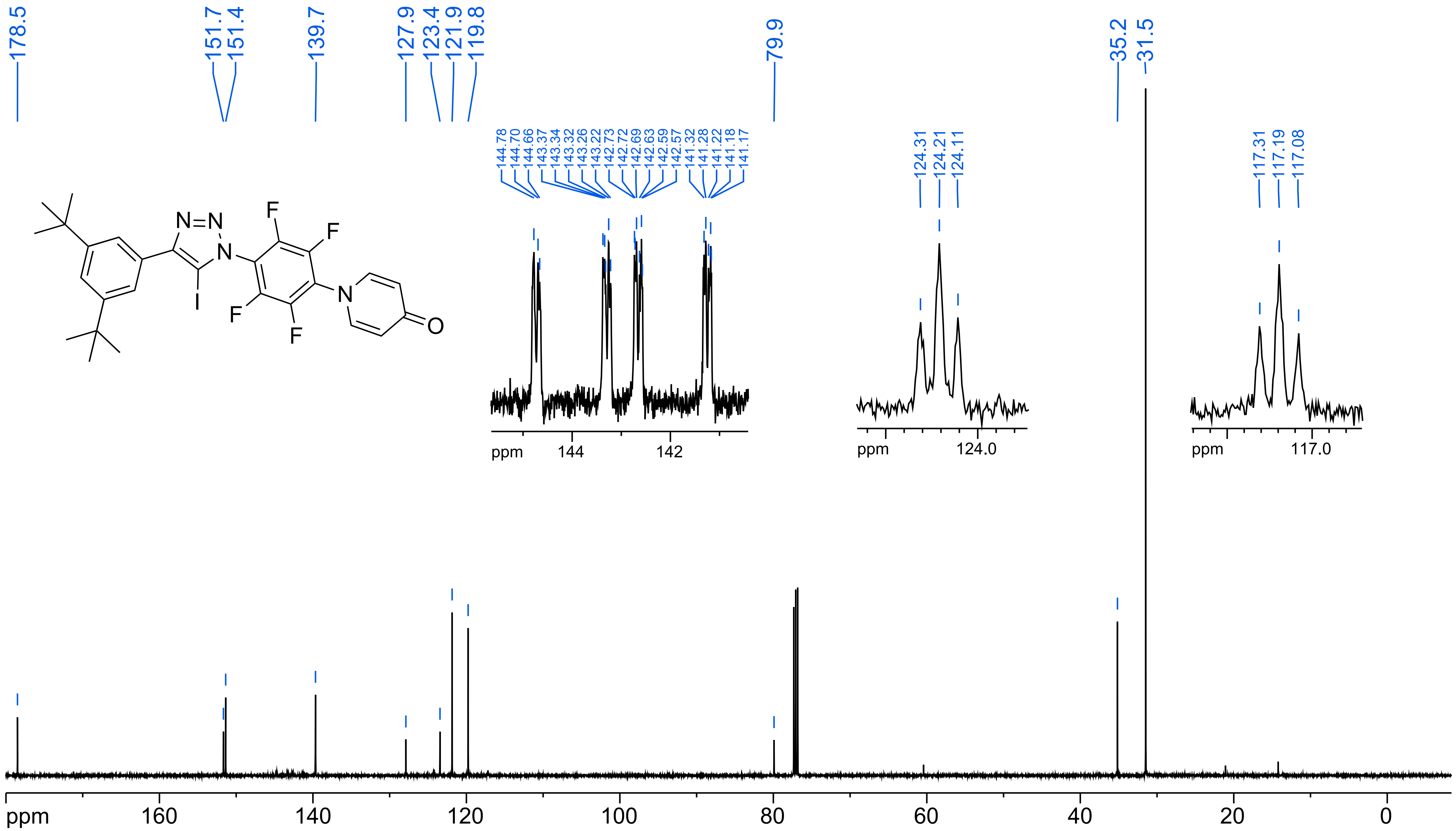

^{13}C NMR (125.7 MHz, RT, $CDCl_3$) spectrum of iodotriazole **1**.

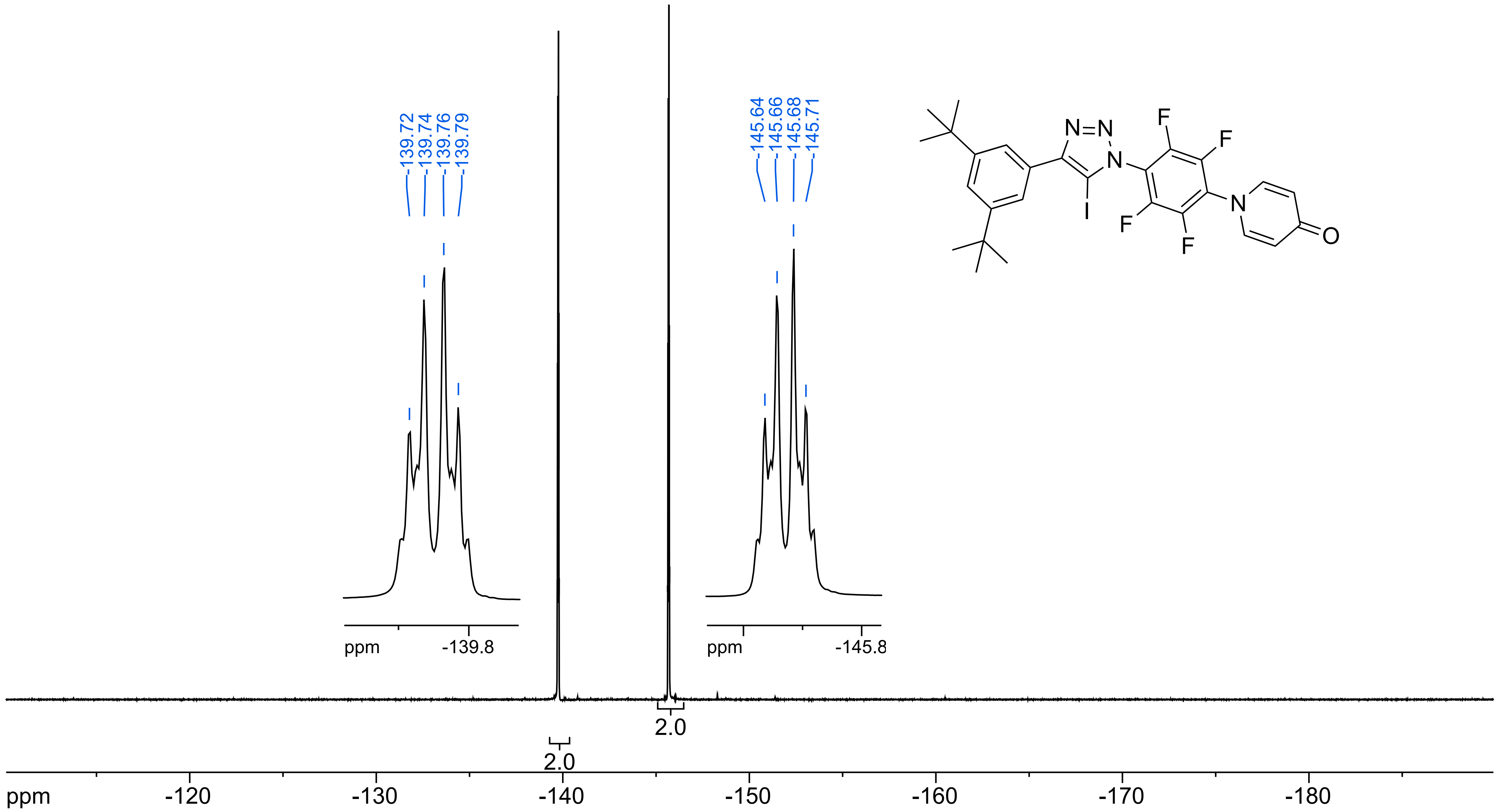

^{19}F NMR (470.6 MHz, RT, $CDCl_3$) spectrum of iodotriazole **1**.

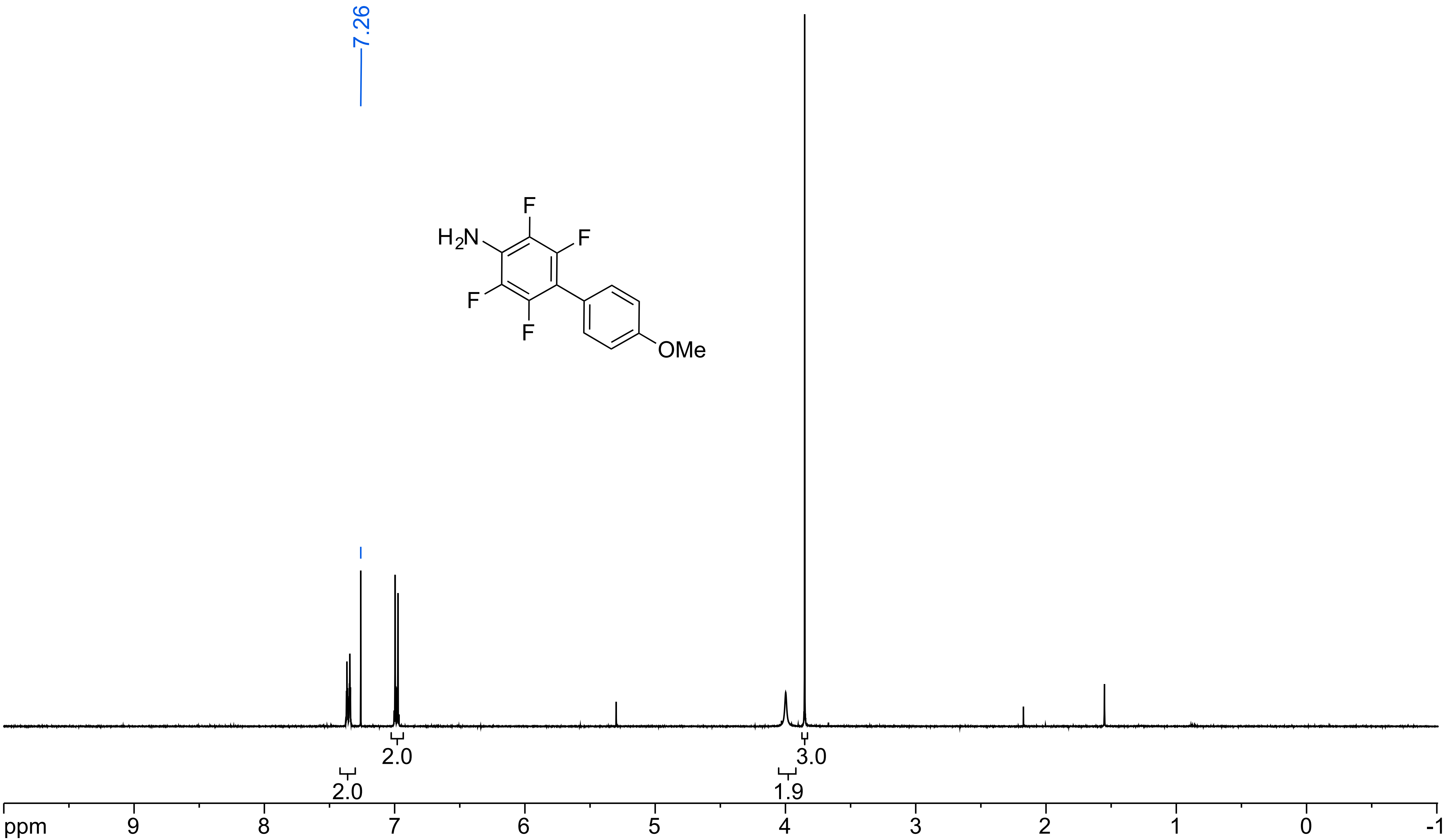

^{1}H NMR (500.1 MHz, RT, $CDCl_3$) spectrum of aniline **S1**.

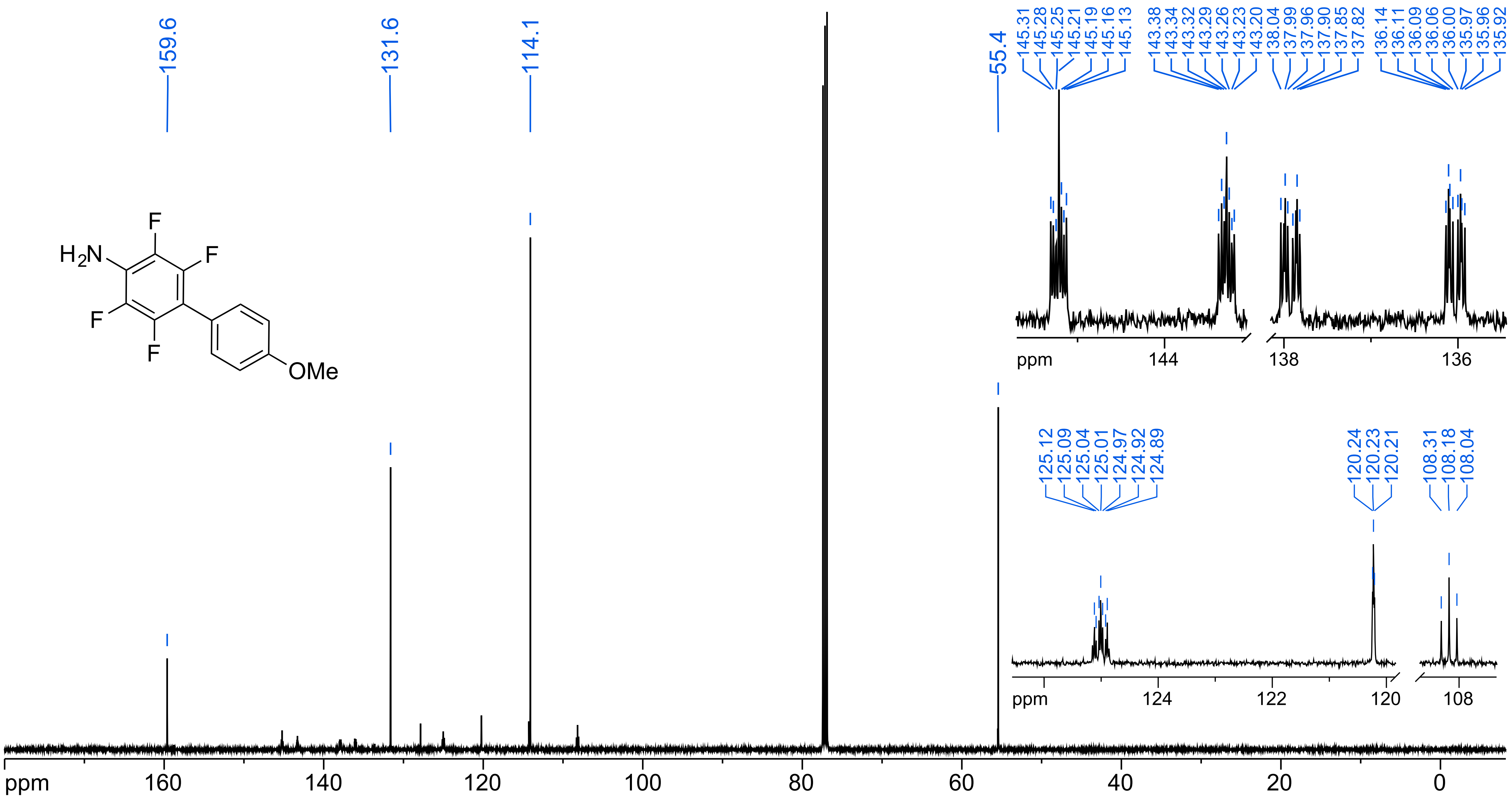

^{13}C NMR (125.7 MHz, RT, $CDCl_3$) spectrum of aniline **S1**.

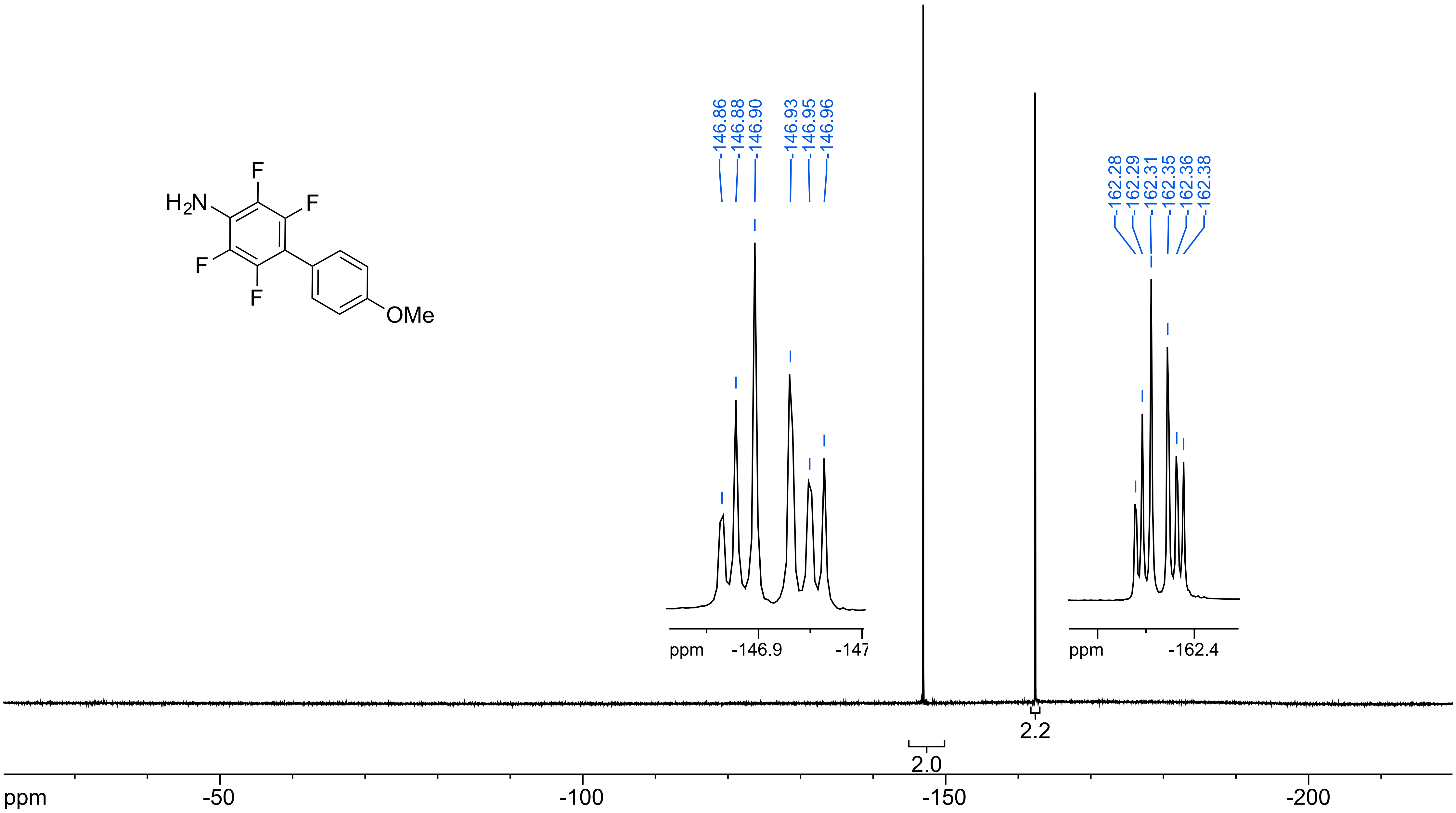

^{19}F NMR (376.4 MHz, RT, $CDCl_3$) spectrum of aniline **S1**.

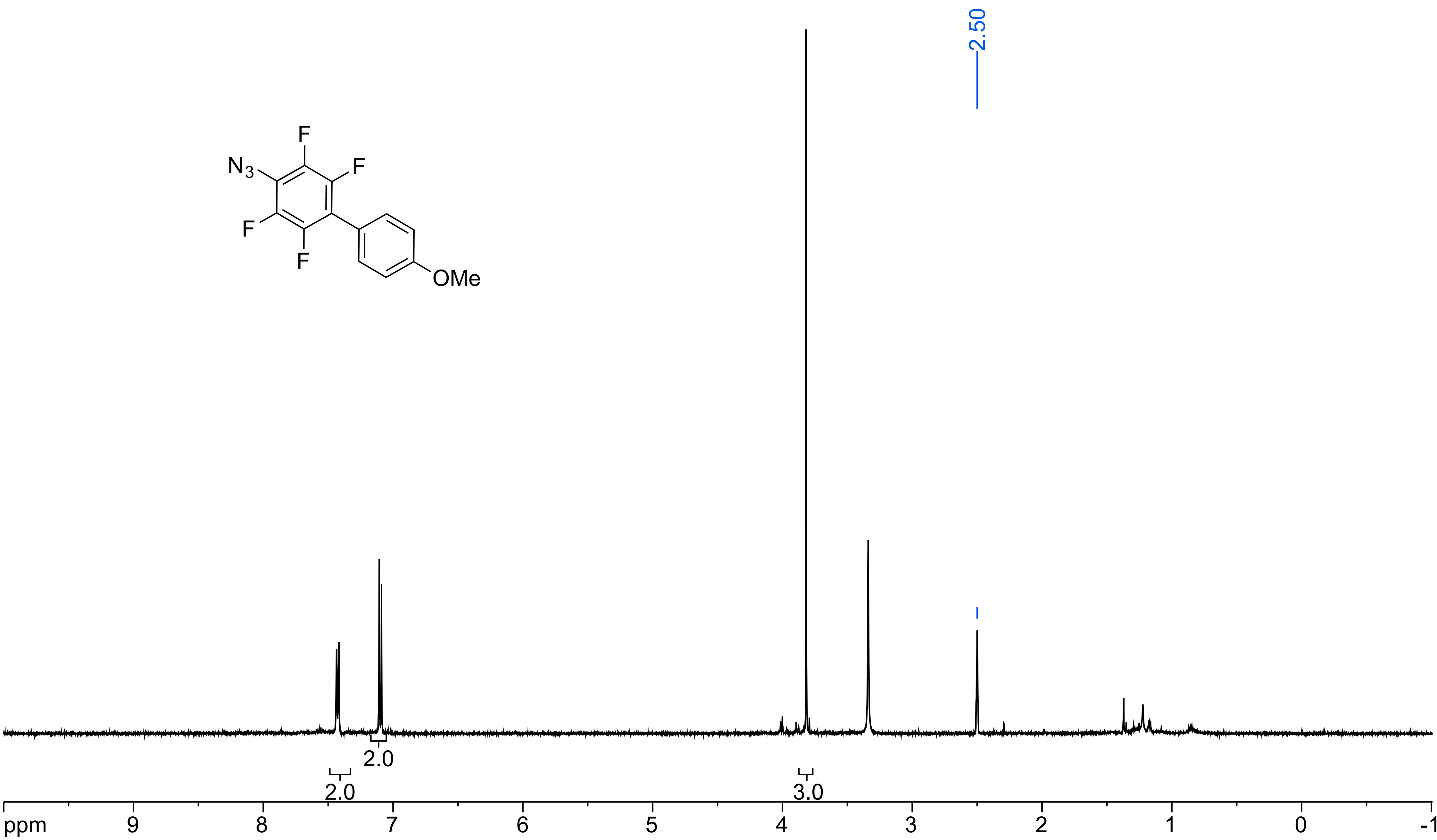

^{1}H NMR (500.1 MHz, RT, d_6-DMSO) spectrum of azide **S2**.

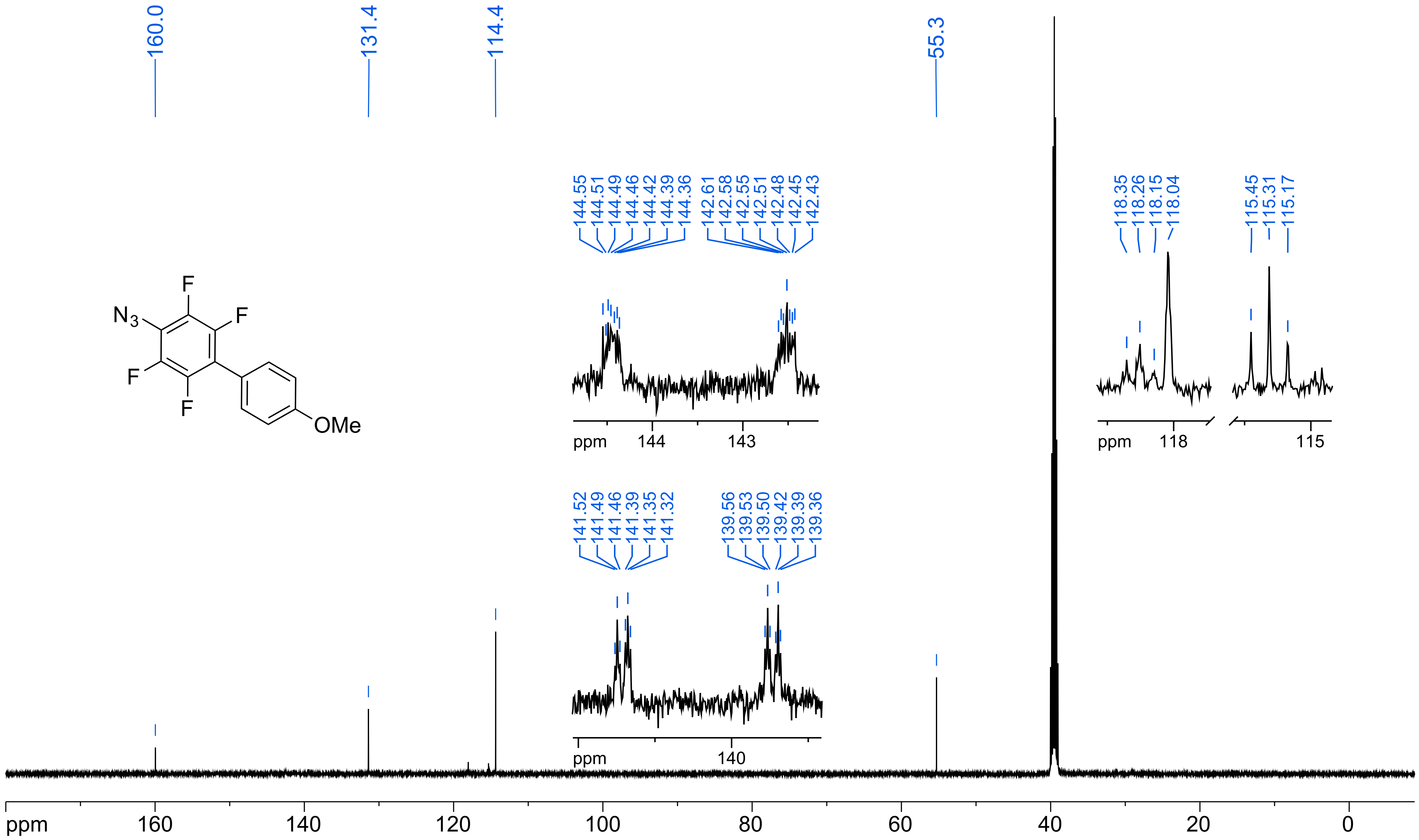

^{13}C NMR (125.7 MHz, RT, d_6-DMSO) spectrum of azide **S2**.

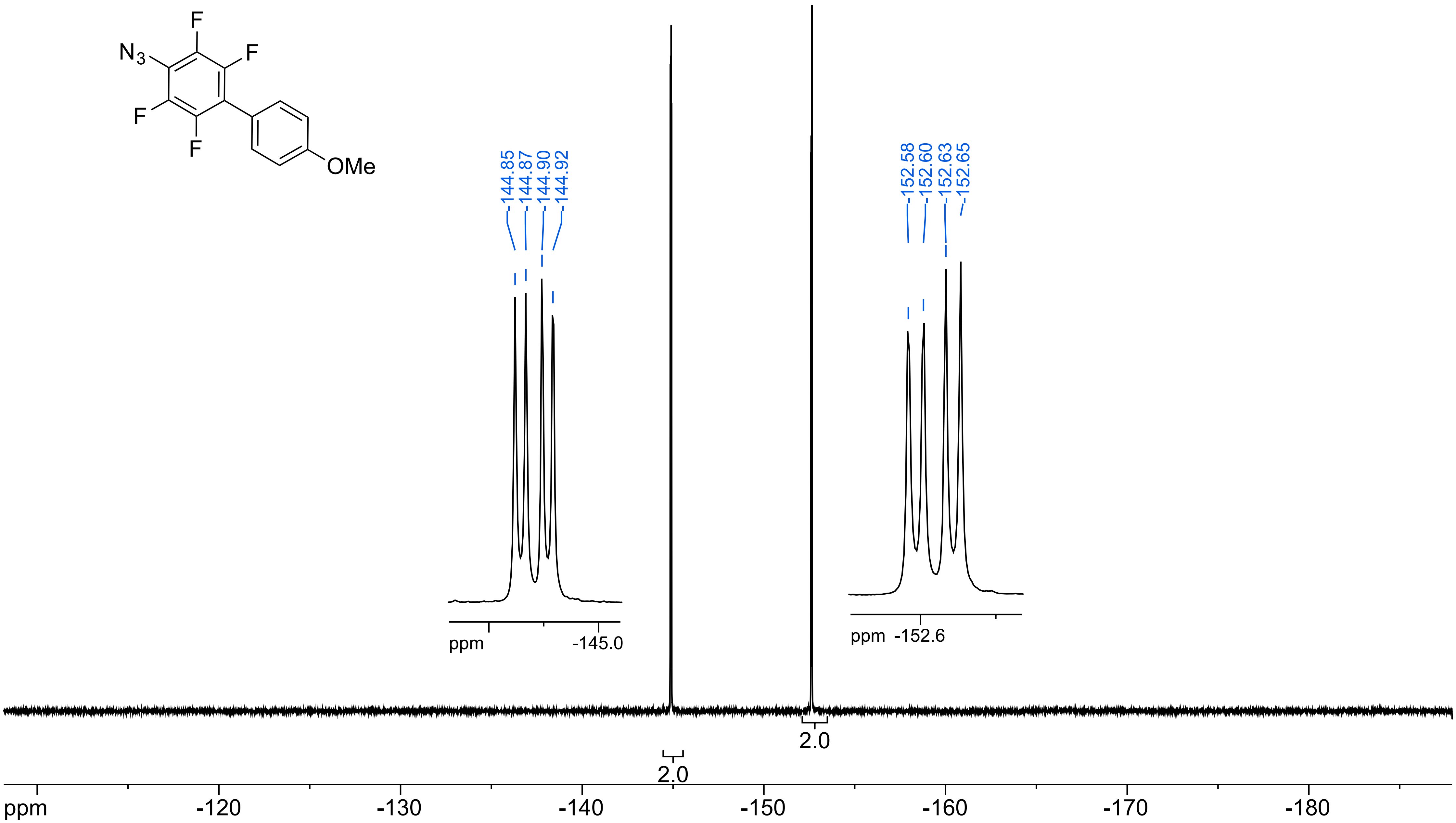

^{19}F NMR (470.6 MHz, RT, d_6-DMSO) spectrum of azide **S2**.

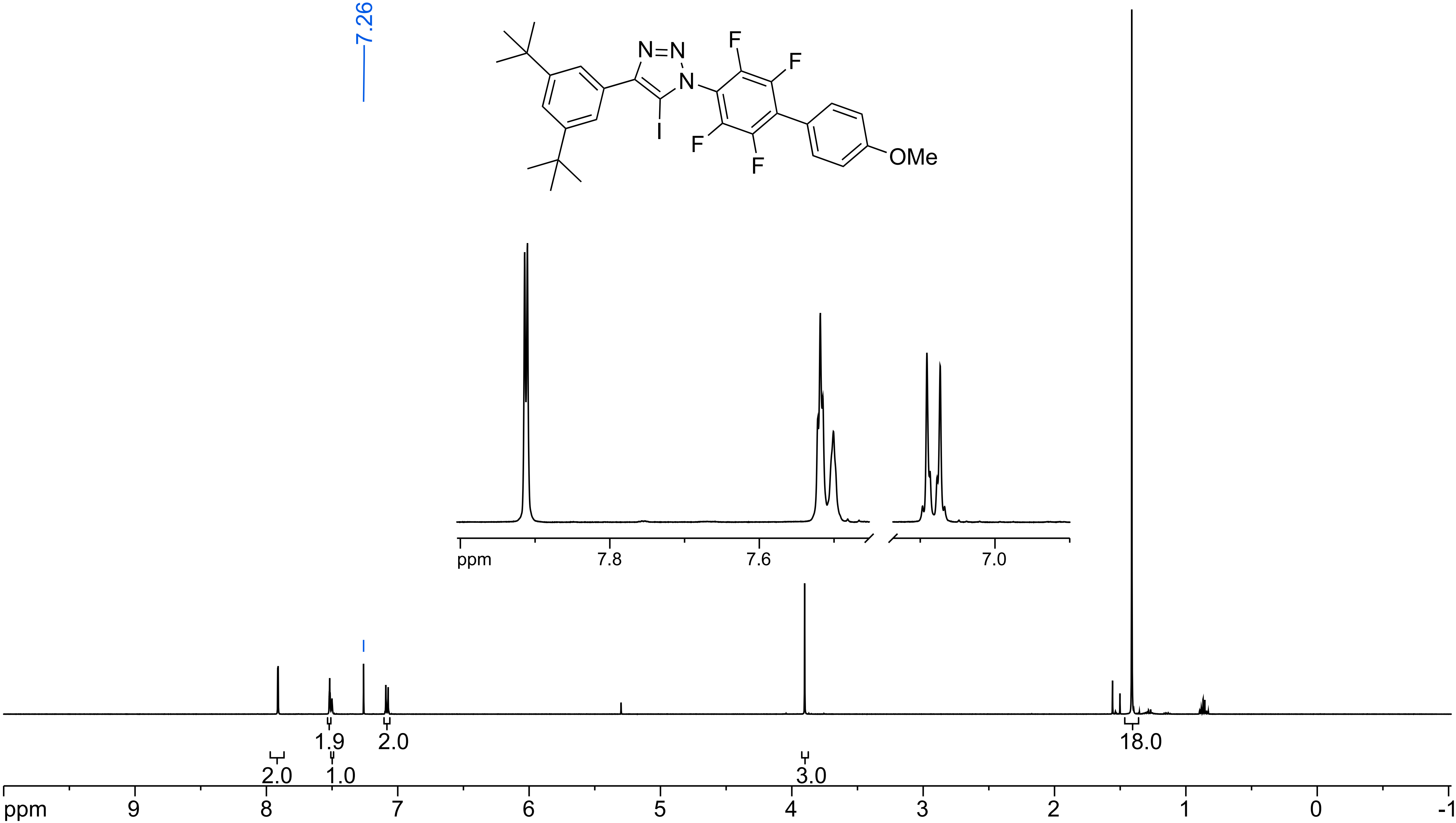

^{1}H NMR (500.1 MHz, RT, $CDCl_3$) spectrum of iodotriazole **S3**.

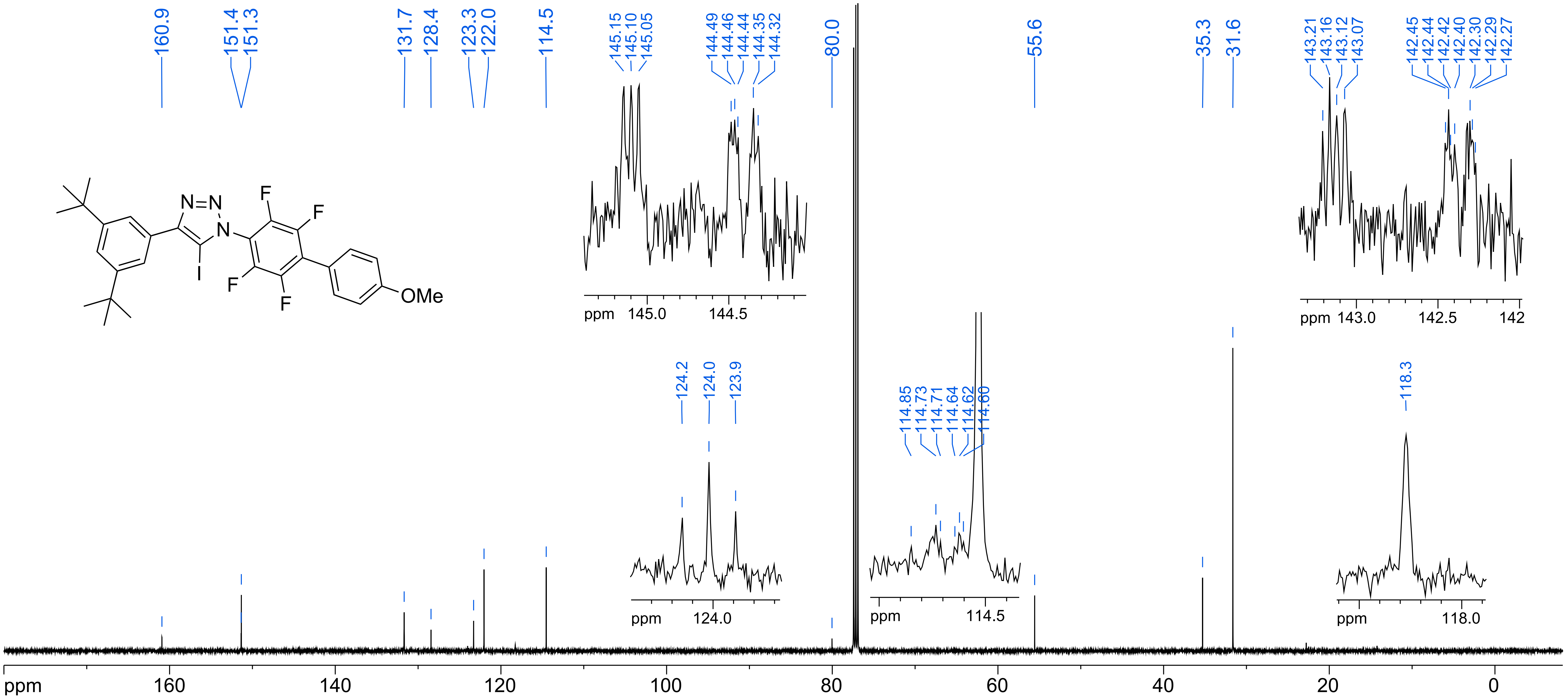

^{13}C NMR (125.7 MHz, RT, $CDCl_3$) spectrum of iodotriazole **S3**.

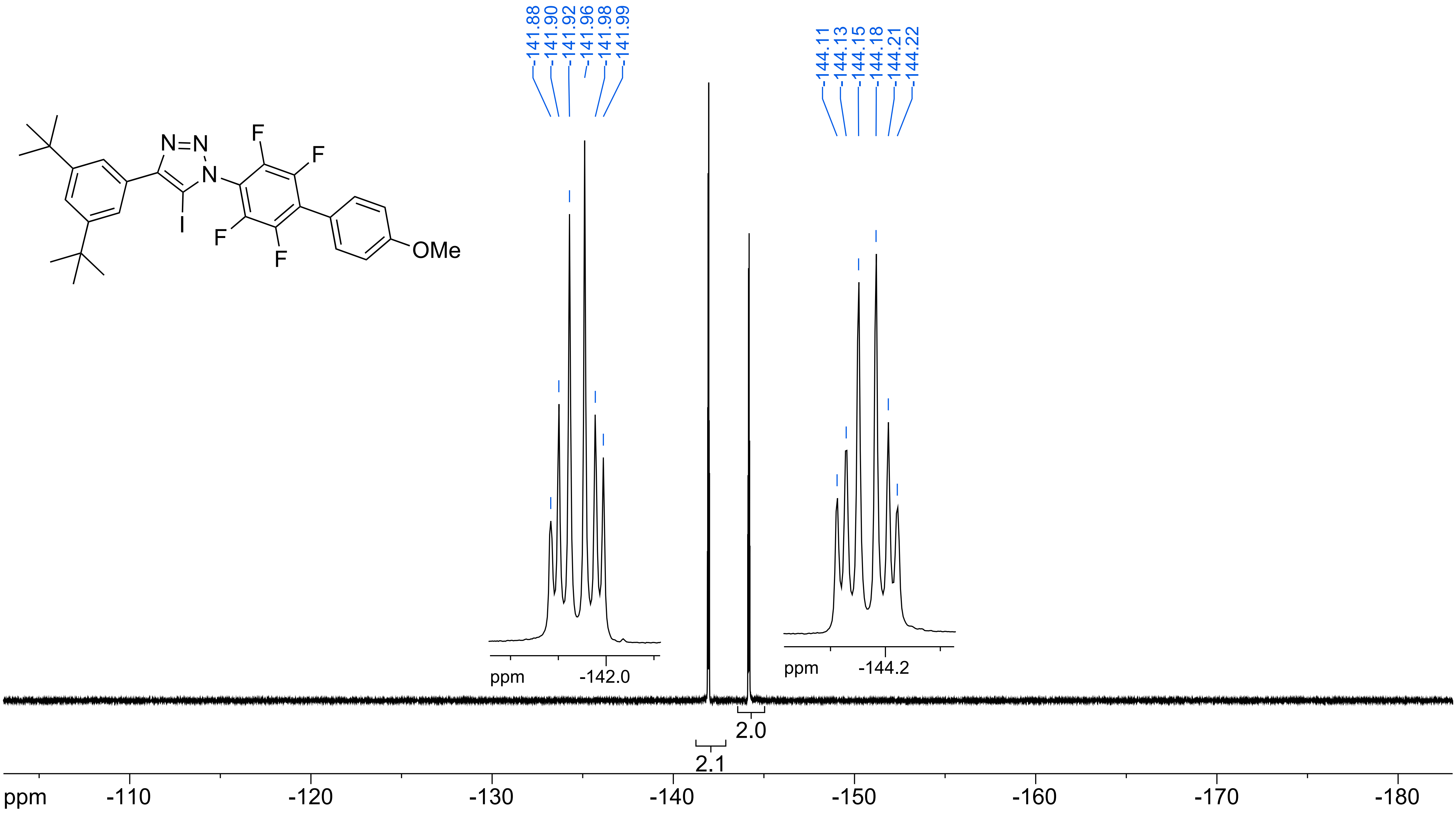

^{19}F NMR (376.4 MHz, RT, $CDCl_3$) spectrum of iodotriazole **S3**.

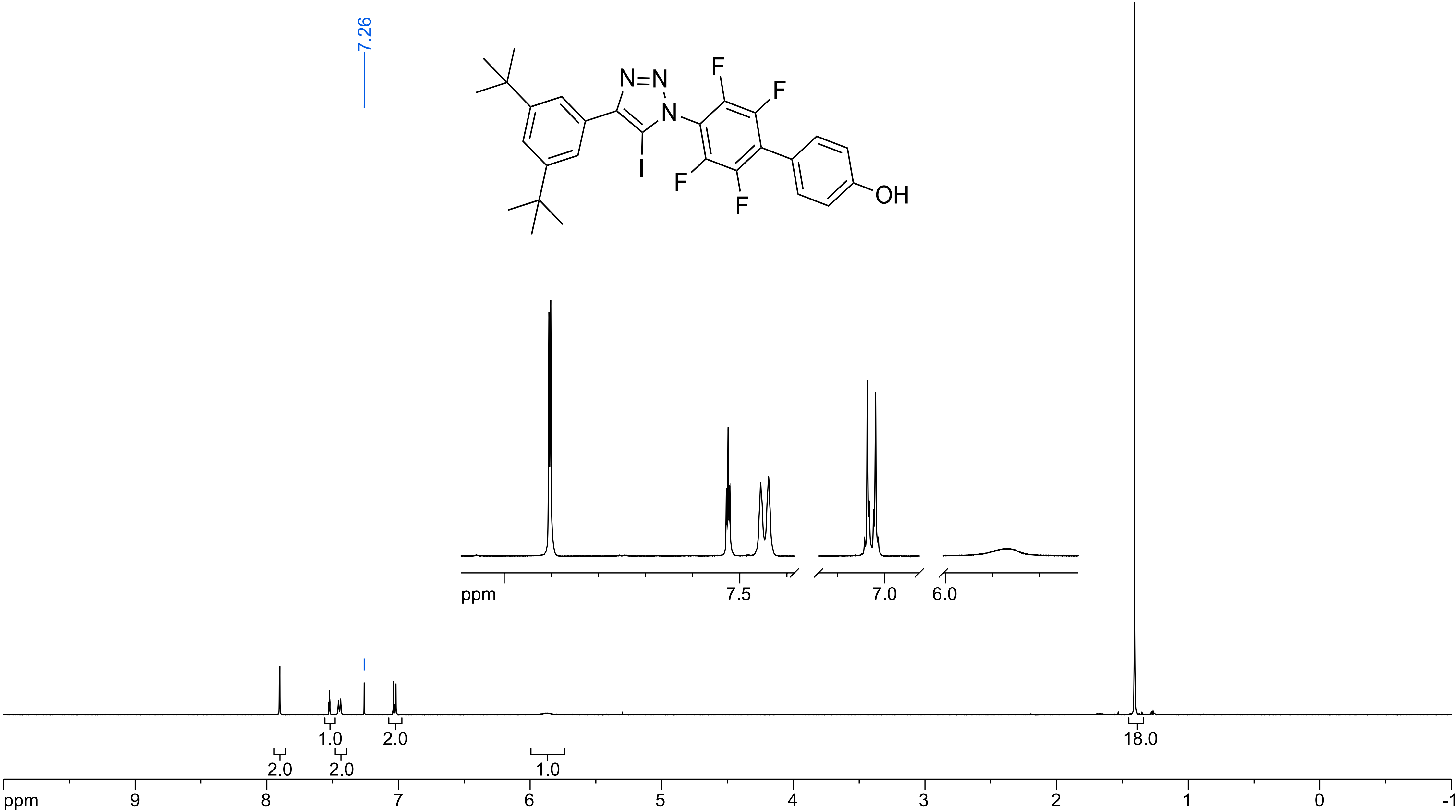

^{1}H NMR (500.1 MHz, RT, $CDCl_3$) spectrum of iodotriazole **2**-H.

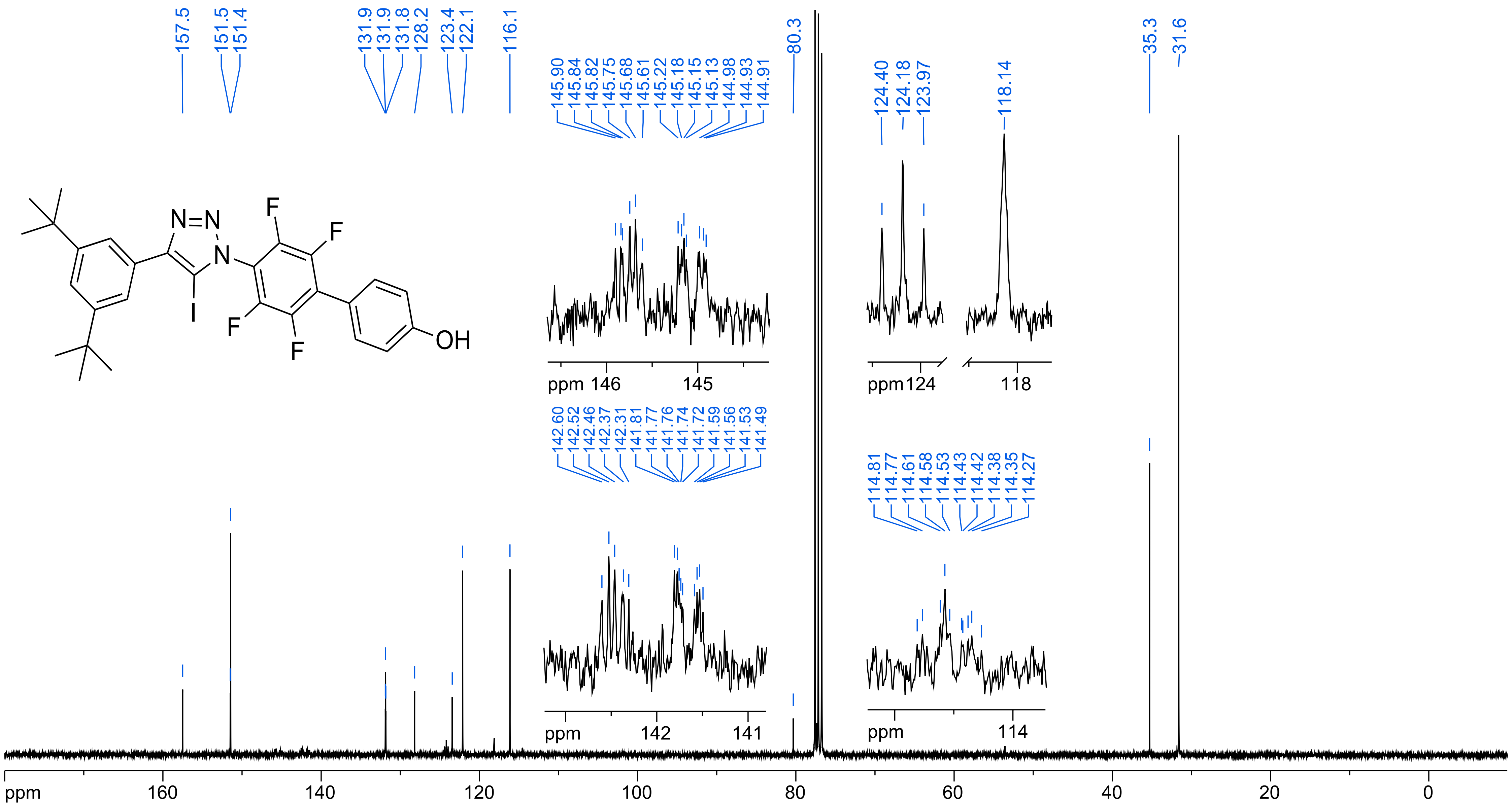

^{13}C NMR (125.7 MHz, RT, $CDCl_3$) spectrum of iodotriazole **2**-H.

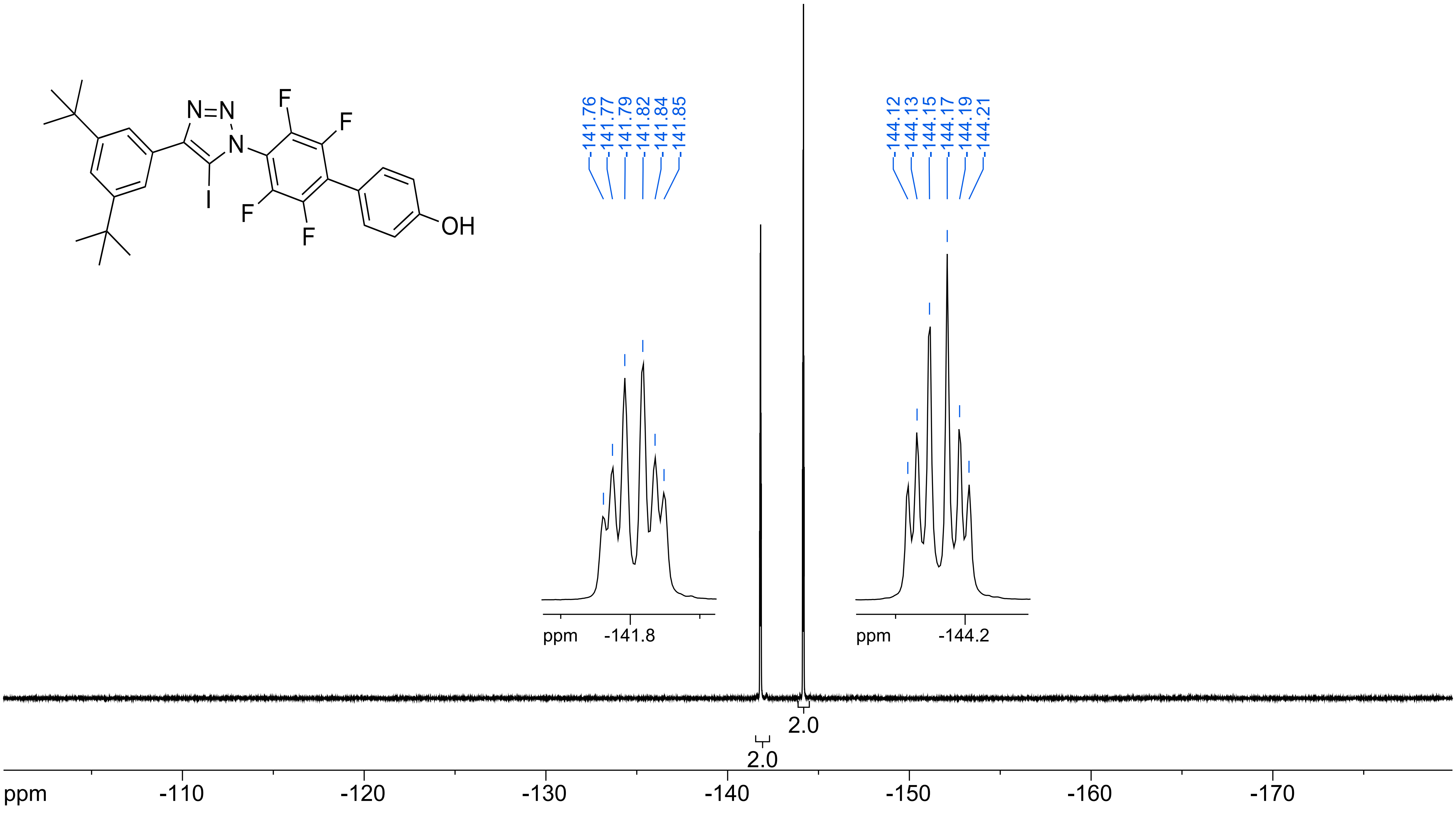

^{19}F NMR (470.6 MHz, RT, $CDCl_3$) spectrum of iodotriazole **2**-H.
